# Manipulation of Wnt/β‐Catenin Signaling by Synthetic Frizzled Agonist and LRP Antagonist in Organoid Cultures and In Vivo

**DOI:** 10.1002/smtd.202500425

**Published:** 2025-03-28

**Authors:** Quanhui Dai, Jiawen Wang, Zihuan Lin, Danni Yu, Hui Yang, Jinsong Wei, Xiaoyu Li, Hao Hu, Chao Ni, Bing Zhao

**Affiliations:** ^1^ State Key Laboratory of Genetic Engineering School of Life Sciences Fudan University Shanghai 200438 China; ^2^ Institute of Organoid Technology Kunming Medical University Kunming 650500 China; ^3^ School of Basic Medical Sciences, The First Affiliated Hospital of Nanchang University, Jiangxi Medical College Nanchang University Nanchang 330031 China; ^4^ Z Lab, bioGenous BIOTECH Shanghai 200438 China

**Keywords:** frizzled agonist, LRP antagonist, organoid culture, signaling manipulation, synthetic key receptor modulator, Wnt/β‐catenin signaling

## Abstract

Wnt/β‐catenin signaling and its dysregulation play critical roles in stem cell fate determination and the pathology of various diseases. However, the application of translated Wnt ligand in regenerative medicine is hampered by its hydrophobicity and cross‐reactivity with Frizzled (FZD) receptors. Here, a synthetic key receptor modulator, the FZD agonist RRP‐pbFn is generated, for high‐efficiency Wnt/β‐catenin signaling activation in the absence of direct binding to LRP5/6. RRP‐pbFn demonstrates superior potency compared to surrogate Wnt, supporting the growth of diverse mouse and human organoids and inducing the expansion of liver and intestine progenitors in vivo. Complementing this, a synthetic LRP antagonist, RRP‐Dkk1c is developed, which exhibits heightened effectiveness in attenuating Wnt/β‐catenin signaling activity compared to Dkk1, thereby abolishing the formation of CT26‐derived colon cancer xenograft in vivo. Together, these two paired key receptor modulators targeting individual type of cell‐surface receptors hold great promise for biomedical research and potential therapeutics.

## Graphical abstract

Harnessing synthetic key receptor modulators, this study presents a breakthrough in Wnt/β‐catenin regulation. The FZD agonist RRP‐pbFn bypasses LRP5/6 to drive organoid expansion and tissue regeneration, while the LRP antagonist RRP‐Dkk1c eradicates tumors. These tools enable precise pathway control, advancing regenerative and cancer therapies. The modular design offers a blueprint for targeting diverse signaling pathways, redefining precision in biomedical intervention.

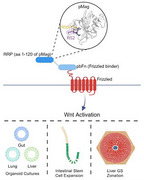

## 1 Introduction

The Wnt/β‐catenin signaling tightly controls embryonic development and tissue homeostasis by regulating stem cell self‐renewal and lineage specification.^[^ ^1^, ^2^ ^]^ WNTs heterodimerize receptors Frizzled (FZD) and low‐density lipoprotein receptor‐related proteins 5/6 (LRP5/6), which triggers the cytoplasmic accumulation of core effector β‐catenin then its nuclear translocation for target genes transcription.^[^ ^3^, ^4^ ^]^ The dysregulation of Wnt/β‐catenin signaling is involved in the pathological processes of various diseases, encompassing developmental defects, cancers, and degenerative disorders.^[^ ^1^, ^5^, ^6^ ^]^ Therefore, manipulation of Wnt/β‐catenin signaling activity in vivo would permit the development of novel therapeutic strategies.

In vitro stem cell expansion and programmed differentiation bring the dawn of regenerative medicine. Since Wnt/β‐catenin signaling governs stem cell self‐renewal, its agonists have been utilized in maintaining stem/progenitor cell expansion. Especially, the newly emerging organoids, derived from adult stem cells through controlled fate determination, have shown great advantages in modeling various development events and diseases for drug discovery, precision medicine and regenerative therapeutics.^[^ ^7^, ^8^, ^9^ ^]^ The design of more potent and stable Wnt modulators will significantly advance stem cell‐related technologies, particularly organoid cultures.

For Wnt/β‐catenin signaling, intracellular agonists bear a greater risk of off‐target effects, such as glycogen synthase kinase‐3 (GSK3) inhibitor CHIR exerting regulatory effects on diverse cellular pathways beyond Wnt/β‐catenin signaling and leading to much broader alterations in global gene expression compared to Wnt3a ligand or its surrogate.^[^ ^10^ ^]^ Extracellular signal ligands bind to specific cell surface receptors to initiate signaling transduction. Therefore, signal receptor agonists exerting the function of natural ligands or antagonists enable the precise regulation of signaling cascades without penetrating cell membrane, which presents great advantages in specificity, safety and delivery compared to intracellular targeting strategies.^[^ ^11^ ^]^ However, the promiscuous interactions arising from the conserved interaction sites between the 19 mammalian WNTs and the 10 FZD subtypes (FZD 1–10) have hindered unequivocally assigning distinct downstream responses to individual FZD subtypes.^[^ ^12^ ^]^ Furthermore, WNTs undergo post‐translational palmitoylation facilitated by the O‐acyltransferase known as Porcupine, which is essential for both WNT secretion and interaction with FZD.^[^ ^13^, ^14^ ^]^ As a result of their palmitoleate groups, WNTs exhibit high hydrophobicity, which necessitates the utilization of detergents for purification and consequently poses challenges in purifying and employing recombinant WNTs.^[^ ^14^ ^]^ Wnt3a conditioned media (CM) is commonly employed as Wnt/β‐catenin signaling activators. Nevertheless, the exact composition of Wnt3a CM is not fully determined, introducing uncontrollable variables in experimental settings.^[^ ^15^ ^]^

Additionally, FZD subtype‐specific next‐generation surrogate Wnt (sWnt/pbF‐Dkk1c, also known as NGS Wnt), which involves linking the protein binder of FZD (pbF, also known as Designed Repeat Protein Binder (DRPB),^[^ ^16^ ^]^ binding to FZD1, 2, 5, 7, 8) to the C‐terminal domain of the human Wnt antagonist Dkk1 (Dkk1c), as well as tetrameric antibody‐based Wnt agonists, have been reported as macromolecules.^[^ ^16^, ^17^, ^18^, ^19^ ^]^ Current surrogate Wnt agonists induce heterodimerization or oligomerization of FZD‐LRP but exhibit weak pathway activation while bypassing the requirement for endogenous Wnts. Additionally, their clinical translation is hindered by manufacturing challenges for endogenous ligands, combined with the suboptimal activity and excessive molecular size of Wnt agonists. Notably, both natural Wnt ligands and existing surrogate Wnts rely on ligand‐receptor complex formation through natural mechanisms. Therefore, it is crucial to engineer a synthetic modulator that targets key receptor without depending on the mechanism of natural ligands.

Inappropriate increase in Wnt receptor activity due to mutational inactivation of the ubiquitin ligases RNF43/ZNRF3 or R‐spondin fusions has recently been identified as a prominent driver of cancer development.^[^ ^20^, ^21^, ^22^ ^]^ The antagonists targeting canonical Wnt receptors hold promise for treating these specific cancer subsets.^[^ ^23^, ^24^, ^25^ ^]^ Thus, the development of synthetic LRP antagonists has great prospects for therapeutic applications.

Here, we report the development of two synthetic key receptor modulators targeting individual type of cell‐surface receptors: the FZD agonist RRP‐pbFn, which consists of RRP (truncation of optogenetic tool pMag) fused with pbFn (N‐terminal domain of pbF),^[^ ^26^, ^27^, ^28^ ^]^ and the LRP antagonist RRP‐Dkk1c. Compared to the improved surrogate Wnt (Dkk1c‐pbF), RRP‐pbFn exhibits distinct characteristics, as it does not directly bind to the LRP5/6 receptor, possesses a lower molecular weight, and activates Wnt/β‐catenin signaling more potently. RRP‐pbFn demonstrates potent support for diverse types of organoid growth and expansion, including small intestine, alveolus, stomach, hepatocyte, and cholangiocyte. Furthermore, we demonstrate the in vivo efficacy of RRP‐pbFn in regulating metabolic liver zonation and promoting adult intestinal proliferation. Synthetic RRP‐Dkk1c manifests superior efficacy in Wnt/β‐catenin signaling inhibition compared to Dkk1, resulting in the inhibition of organoid growth and effective suppression of tumor growth in the CT26 xenograft model. Notably, these key receptor modulators, with their artificially designed water‐soluble properties and small molecular weight, hold great promise for advancing research and translational applications in regenerative medicine, as well as providing an adaptable approach for developing key receptor modulators for other signaling pathways.

## 2 Results

### 2.1 Generation of a Synthetic Frizzled Agonist for High‐Efficiency Wnt/β‐Catenin Signaling Activation

The precise spatial and temporal control of cell signaling has widespread application in regenerative medicine and the study of tissue development.^[^ ^29^, ^30^ ^]^ We initially aimed to develop a photoswitch‐controlled sWnt for spatiotemporal control of Wnt/β‐catenin signaling. Engineered photoswitches, Magnets (pMag/nMag), which undergo light‐induced heterodimerization, have been utilized to create an optogenetic tool.^[^ ^26^ ^]^ We employed this light‐activated Magnets dimerization system by fusing splits of the sWnt fragments (pbF/Dkk1c) with the pMag/nMag (Figure S1A, Supporting Information). Theoretically, split sWnt fragments could be functionally restored via the light‐induced pMag/nMag heterodimerization (Figure S1B, Supporting Information). Variants of sWnt/Magnets halves were transfected individually or in combination into human embryonic kidney 293T (HEK 293T) cells along with TOP‐Flash and *Renilla* reporters. The luciferase activity was subsequently determined with or without illumination. However, the pMag/nMag‐based sWnt system showed no enhanced TOP‐Flash signal after illumination, suggesting that splitting sWnt into pbF and Dkk1c was unsuitable for Magnets photoswitch‐controlled sWnt activity (Figure S1C, Supporting Information). Surprisingly, pMag‐pbF alone, even without a combination or blue light, induced significant and robust Wnt activation (Figure S1C, Supporting Information).

**Figure 1:**
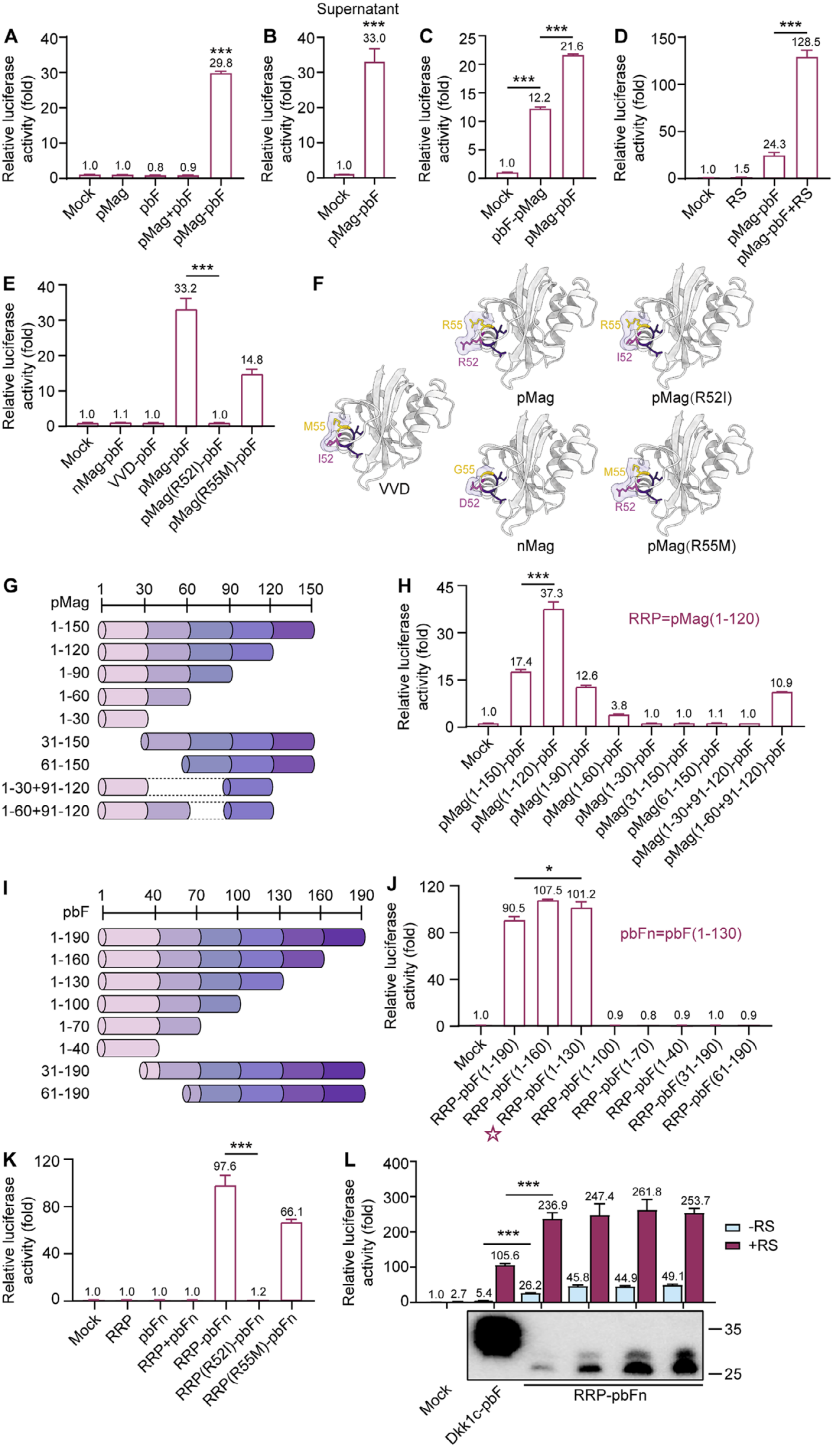
Generation of a synthetic Frizzled agonist for high‐efficiency Wnt/β‐catenin signaling activation. A) TOP‐Flash luciferase reporter assay showing the Wnt activity induced by overexpression of pMag‐pbF or control proteins. The empty vector (mock) along with reporter plasmids was used as a negative control. B) TOP‐Flash luciferase reporter assay showing activation of Wnt/β‐catenin signaling by 50% pMag‐pbF CM. C) TOP‐Flash luciferase reporter assay showing the Wnt activity induced by overexpression of pMag‐pbF (pMag fused to the N‐terminal of pbF) and pbF‐pMag (pbF fused to the N‐terminal of pMag). D) 250 ng mL^−1^ R‐spondin1 potentiated the Wnt activity induced by pMag‐pbF overexpression. E) The Wnt activity induced by nMag‐pbF, VVD‐pbF, pMag(R52I)‐pbF, and pMag(R55M)‐pbF overexpression. F) The structure of VVD, pMag, nMag, pMag(R52I), and pMag(R55M) with different amino acid residues. VVD has amino acids I/M at positions 52 and 55, while pMag and nMag have R/R and D/G, respectively. Mutated pMag(R52I) and pMag(R55M) have I/R and R/M, respectively. Surface of each protein is showed as light purple patches. G) Schematic representation of the full‐length and various truncated pMag. The numbers listed on the left represent the position of the site of truncation based on a full‐length pMag. H) The Wnt activity induced by overexpression of the variants of full‐length or truncated pMag fused with pbF. RRP = pMag(1‐120). I) Schematic representation of the full‐length and various truncated pbF. The numbers listed on the left represent the position of the site of truncation based on a full‐length pbF. J) The Wnt activity induced by overexpression of RRP fused with the variants of full‐length or truncated pbF. pbFn = pbF(1‐130). K) The Wnt activity induced by overexpression of RRP, pbFn, RRP+pbFn, RRP‐pbFn, and mutated RRP(R52I)‐pbFn and RRP(R55M)‐pbFn. L) Overexpressing RRP‐pbFn induced higher dose‐dependent Wnt/β‐catenin signaling activation than the improved surrogate Wnt (Dkk1c‐pbF) with or without 250 ng mL^−1^ R‐spondin1. The bottom panel is detection of the improved surrogate Wnt (Dkk1c‐pbF) and RRP‐pbFn in supernatant by Western blot using Anti‐Myc‐antibody after overexpression. All TOP‐Flash data represent mean ± SD; *n* = 3 independent experiments. *p < 0.05; **p < 0.01; ***p < 0.001.

After observing the strong Wnt activation triggered by the pMag‐pbF fusion protein, we proceeded to test whether pMag and pbF could activate Wnt/β‐catenin signaling on their own or in combination. As a consequence, only the pMag‐pbF fusion protein showed a 29.8‐fold inducible effect on TOP‐Flash signal, whereas neither pMag alone, pbF alone, nor their combination exhibited such effect (**Figure** **1**A). All proteins were expressed and secreted into supernatant with comparable quantity (Figure S2A, Supporting Information). 50% pMag‐pbF conditioned media (CM) also activated the Wnt/β‐catenin signaling (Figure 1B). Interestingly, we observed that orientation had an impact on activity for pMag‐pbF fusion protein, where pbF‐pMag (pbF fused to the N‐terminal of pMag) exhibited lower activity than pMag‐pbF (pMag fused to the N‐terminal of pbF) in inducing Wnt activation (Figure 1C). In addition, pMag‐pbF showed synergy with Wnt potentiator R‐spondin1,^[^ ^31^ ^]^ demonstrated by the enhanced expression of the TOP‐Flash reporter in HEK 293T cells (Figure 1D). The substitution of the amino acid residue Ile52 and Met55 of a blue light photoreceptor derived from the filamentous fungus, Vivid (VVD) with R/R (I52R/M55R) or D/G (I52D/M55G) gave rise to pMag and nMag, respectively.^[^ ^26^ ^]^ Subsequently, we investigated the capacity of the nMag‐pbF and VVD‐pbF fusion proteins to induce Wnt/β‐catenin signaling, but no activation was observed (Figure 1E). Structural analysis of pMag, VVD, and nMag proteins revealed that structural variations were restricted to specific amino acid residues 52 and 55 (Figure 1F). Based on this observation, we hypothesized that R52 and R55 residues of pMag were critical for the agonist activity of pMag‐pbF. To confirm this, we generated pMag variants with substitutions of R52 with I or R55 with M, resulting in pMag(R52I) and pMag(R55M), respectively (Figure 1F), which were subsequently fused with pbF. Our findings confirmed the hypothesis, as pMag(R55M)‐pbF showed decreased activity, whereas pMag(R52I)‐pbF had no activity when compared to pMag‐pbF (Figure 1E).

The utility and druggability of synthetic agonists depend heavily on the molecular weight. We then explored the shortest variant with highest‐efficiency Wnt/β‐catenin signaling activation by truncating pMag and pbF separately. Truncated variants of pMag (150 aa in total) and pbF (190 aa in total) were generated by stepwise deleting 30 aa from either the N or C‐terminal (Figure 1G,I). We performed western blot to ensure the expression of truncated variants of pMag fusion with pbF, and the results indicated the comparable quantity of fusion proteins (Figure S2B, Supporting Information). Following the first 120 aa of pMag (pMag(1‐120)) fusion with pbF, pMag(1‐120)‐pbF exhibited the strongest Wnt activation, while the first 90 aa of pMag fusion with pbF (pMag(1‐90)‐pbF) showed decreased activity compared to pMag‐pbF (Figure 1H). Although the first 60 aa of pMag fusion with pbF (pMag(1‐60)‐pbF) showed weak activity, the first 60 plus 91–120 aa of pMag fusion with pbF (pMag(1‐60+91‐120)‐pbF) restored the activity. Thus, we named pMag(1‐120) as RRP. Subsequently, the western blot results indicated the comparable expression levels of truncated fusions of RRP‐pbF (Figure S2C, Supporting Information). Following the first 130 aa of pbF (pbF(1‐130)) fusion with RRP, RRP‐pbF(1‐130) displayed more potent activation than RRP‐pbF(1‐190) (Figure 1J), and pbF(1‐130) was named N‐terminal domain of pbF (pbFn). Furthermore, RRP‐pbFn‐induced Wnt activation remained unchanged after blue light illumination (Figure S3A, Supporting Information). Using mutagenesis of RRP within pMag(R52I) and pMag(R55M), we observed that RRP(R55M)‐pbFn displayed diminished activity, whereas RRP(R52I)‐pbFn showed no activity, consistent with the behavior of pMag‐pbF (Figure 1K). Moreover, pbFn and RRP(R52I)‐pbFn inhibited the activation of Wnt/β‐catenin signaling induced by RRP‐pbFn (Figure S3B, Supporting Information).

### 2.2 RRP‐pbFn Activates Wnt/β‐Catenin Signaling More Potently than Improved Surrogate Wnt

We then set out to determine whether FZD agonist RRP‐pbFn exhibits superior capacity for activating Wnt/β‐catenin signaling. Previously reported sWnt (pbF‐Dkk1c) induced Wnt/β‐catenin signaling activity,^[^ ^16^ ^]^ and when the LRP6 binder was fused to the N‐terminus of the FZD binder resulting in higher activity in comparison with the other orientation.^[^ ^17^ ^]^ Therefore, we compared the activity of improved surrogate Wnt (Dkk1c‐pbF) with RRP‐pbFn in a dose‐dependent manner using Wnt reporter assays in HEK 293T cells. RRP‐pbFn induced dose‐response and stronger Wnt/β‐catenin signaling even at low concentration than improved surrogate Wnt with or without R‐spondin1 (Figure 1L). Additionally, RRP‐pbFn exhibited a smaller molecular weight in comparison to improved surrogate Wnt (Figure 1L). To analyze β‐catenin stabilization, we performed protein analysis, which revealed that RRP‐pbFn induced significantly stronger stabilization of β‐catenin compared to Dkk1c‐pbF (Figure S4A, Supporting Information). In summary, RRP‐pbFn, a water‐soluble synthetic FZD agonist with a smaller molecular weight, activates Wnt/β‐catenin signaling much more potently than improved surrogate Wnt. These results suggest that targeting FZD might be a promising strategy to discover next‐generation Wnt agonist.

### 2.3 RRP‐pbFn Facilitates the Growth of Various Mouse and Human Organoids

**Figure 2:**
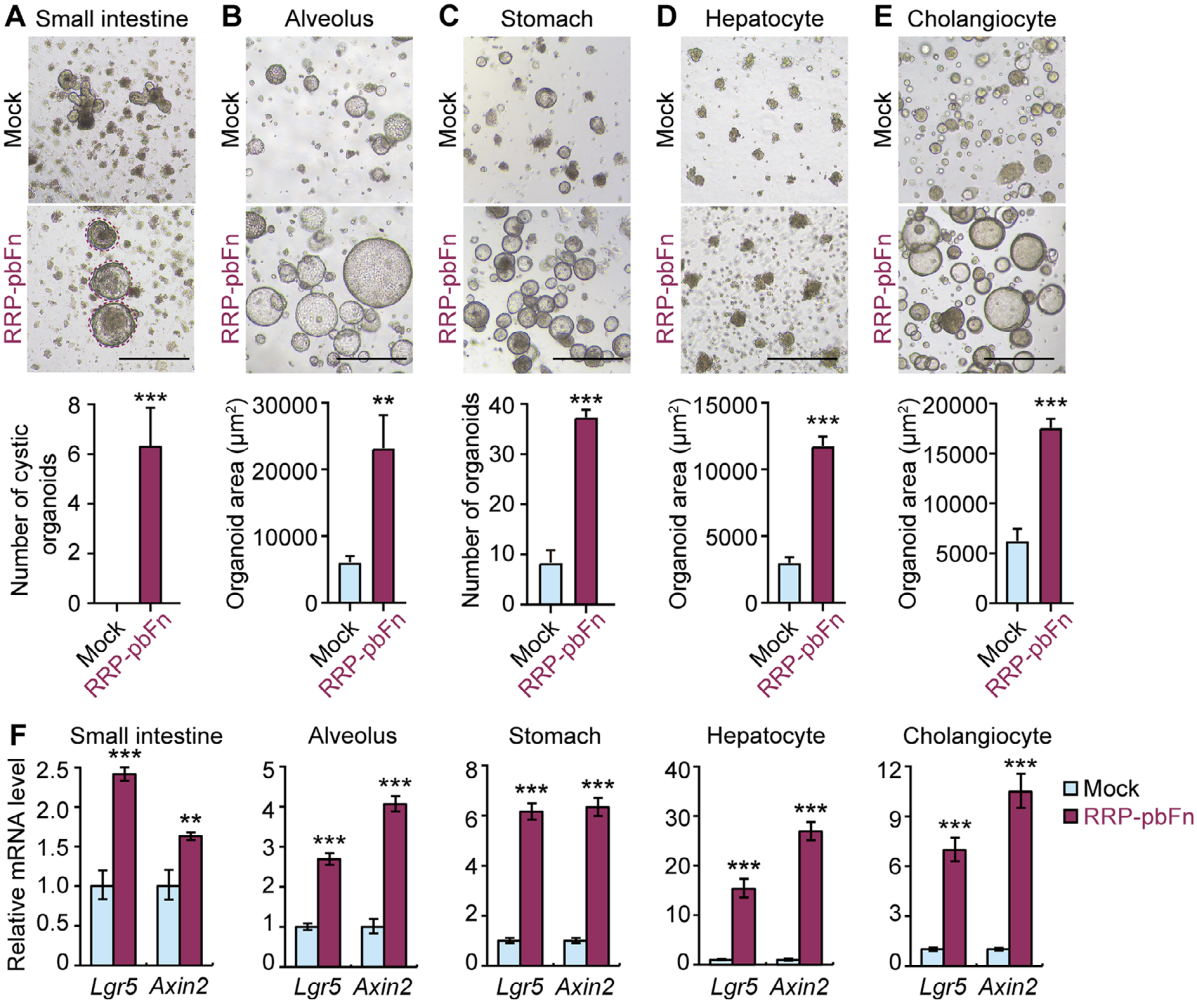
RRP‐pbFn facilitates the growth of various mouse and human organoids. A–E) Representative bright‐field images of mouse organoids derived from small intestine (A), alveolus (B), stomach (C), and hepatocyte (D) expanded in culture media, and human cholangiocyte (E) expanded in culture media containing 3 µM IWP‐2, and supplemented with or without 200 ng mL^−1^ recombinant RRP‐pbFn. The bottom panels are quantification of the organoid number (small intestine, stomach) or organoid area (alveolus, hepatocyte, and cholangiocyte). Data represent mean ± SD, and *n* = 3 for each organoid type. The scale bar represents 500 µm. F) qRT‐PCR analysis of gene expression of Wnt/β‐catenin signaling target genes. Data represent mean ± SD. *p < 0.05; **p < 0.01; ***p < 0.001.

As Wnt/β‐catenin signaling plays a dominant role in tissue stem cell self‐renewal thus long‐term maintenance in vitro, its ligand or surrogate is widely employed in the culture system of stem/progenitor cells even organoids.^[^ ^16^, ^32^, ^33^ ^]^ We assessed the potential of RRP‐pbFn to function as a next‐generation Wnt/β‐catenin agonist in supporting organoid cultures. Multiple types of organoids were generated from mouse small intestine, alveolus, stomach, hepatocyte, and human cholangiocyte, then cultured in the presence or absence of recombinant RRP‐pbFn. The basal media‐cultured intestinal organoids displayed normal morphology as previously described,^[^ ^33^ ^]^ whereas RRP‐pbFn‐supported organoids became thin‐walled cystic structures, indicating more proliferation (**Figure** **2**A). RRP‐pbFn demonstrated tremendous improvement of the number of stomach‐derived organoids and the area of alveolus‐, hepatocyte‐, and human cholangiocyte‐derived organoids (Figure 2B–E). Moreover, this was strongly paralleled by the enhanced transcription level of Wnt target genes *Lgr5* and *Axin2* by RRP‐pbFn (Figure 2F). RRP‐pbFn and Dkk1c‐pbF proteins were then administered to cholangiocyte organoids. Compared to Dkk1c‐pbF, RRP‐pbFn showed a significantly greater improvement in the organoid area (Figure S4B, Supporting Information). Collectively, these findings demonstrate that RRP‐pbFn supports the growth of various organoid models.

### 2.4 RRP‐pbFn Induces the Expansion of Liver and Intestine Progenitors Through Manipulating Wnt/β‐Catenin Signaling In Vivo

**Figure 3:**
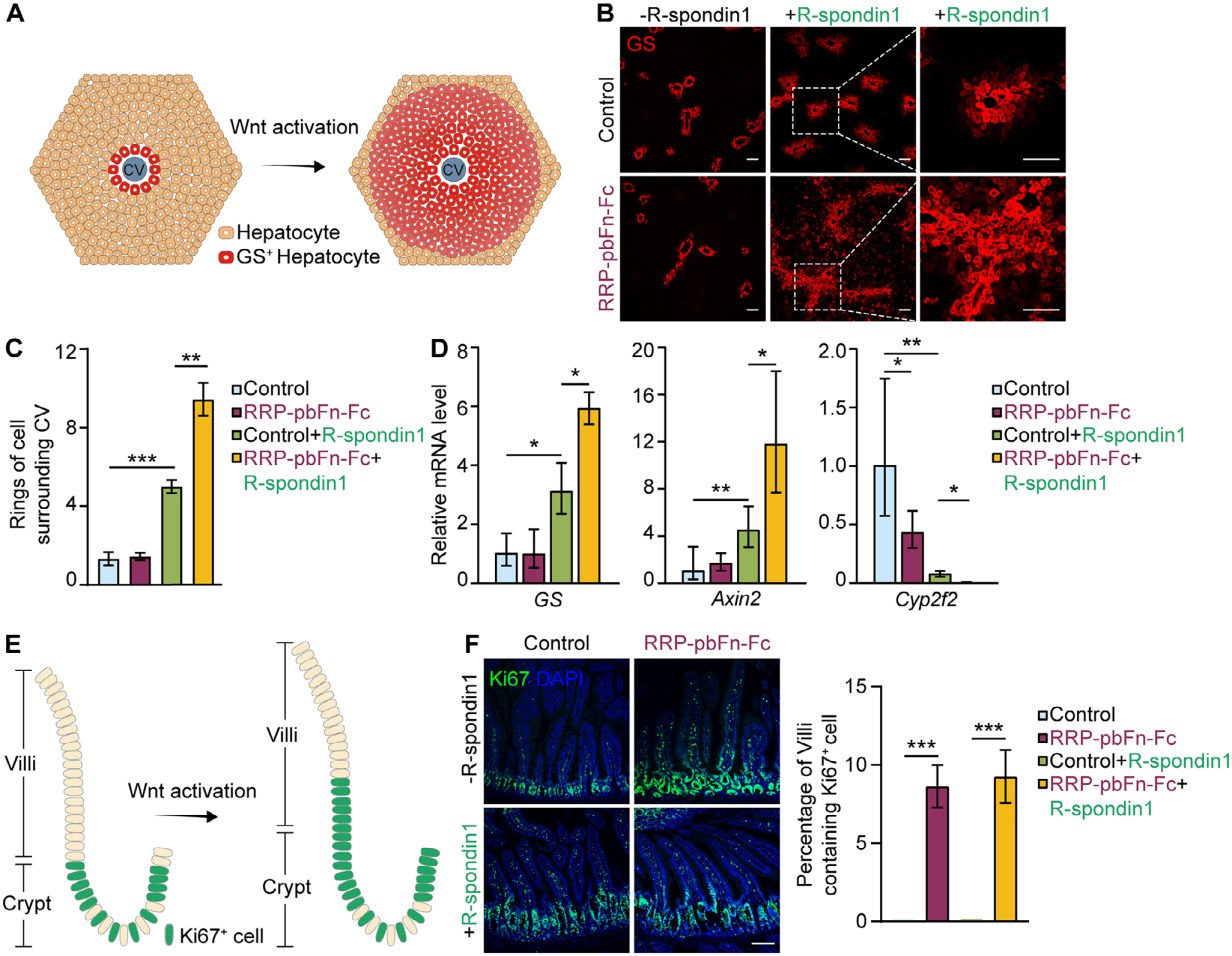
RRP‐pbFn induces the expansion of liver and intestine progenitors through manipulating Wnt/β‐catenin signaling in vivo. A) Scheme depicting activation of Wnt/β‐catenin signaling regulating liver zonation. B) Representative images of GS (pericentral marker) immunofluorescence staining of livers from mice following AAV and R‐spondin1 administration. The scale bar represents 100 µm. C) Quantification of the rings of GS^+^ hepatocytes around the central vein. D) qRT‐PCR analysis of Wnt target genes *GS* and *Axin2*, periportal marker *Cyp2f2* of livers from mice received AAV and R‐spondin1 administration. E) Scheme depicting activation of Wnt/β‐catenin signaling promoting adult intestinal proliferation. F) Representative images of Ki67 immunofluorescence staining of jejunum from mice following AAV and R‐spondin1 administration. DAPI staining is represented in blue. The scale bar represents 100 µm. The right panel is quantification of percentage of Villi containing Ki67^+^ cells. Data represent mean ± SD. *n* = 3 mice per group. *p < 0.05; **p < 0.01; ***p < 0.001.

As FZD agonist RRP‐pbFn exhibits high‐efficiency and stable ability of regulating cell fate determination in the near‐physiology organoids, we then moved to testify whether RRP‐pbFn works in vivo. The physiological processes of adult liver zonation and intestinal stem cell expansion represent archetypal Wnt‐responsive events.^[^ ^1^, ^16^, ^34^ ^]^ To assess the in vivo activity of RRP‐pbFn, we utilized adeno‐associated virus 8 (AAV‐8), a delivery system that can infect hepatocytes in liver.^[^ ^35^ ^]^ RRP‐pbFn was fused with mouse IgG Fc fragment (Fc) to prolong the circulating half‐life. Subsequently, mice were injected with AAV‐8 expressing RRP‐pbFn‐Fc or Fc as a negative control, followed by injection with or without recombinant R‐spondin1 (Figure S5A, Supporting Information). Validation of AAV‐mediated RRP‐pbFn‐Fc expression in mouse liver was confirmed by qRT‐PCR analysis (Figure S5B, Supporting Information). In the liver, WNTs are expressed in the vicinity of the central vein (CV) and the activation of Wnt/β‐catenin signaling triggers the pericentral gene expression program in adjacent hepatocytes,^[^ ^36^ ^]^ including *glutamine synthetase* (*GS*), consequently perturbing metabolic zonation (**Figure** **3**A). We observed that single treatment with AAV‐RRP‐pbFn‐Fc did not significantly affect liver GS expression, but combinatorial treatment with recombinant R‐spondin1 induced a synergistic expansion of the GS‐expressing pericentral region (Figure 3B,C), and up‐regulated *GS* and *Axin2* while repressing the periportal marker *Cyp2f2* mRNA compared with control mice (Figure 3D).

In the small intestine, Wnt/β‐catenin signaling activation stimulates crypt hyperplasia and expansion of mitotic Ki67^+^ epithelium (Figure 3E). Upon intravenous injection, AAV‐8 was capable of infecting mouse liver hepatocytes, resulting in persistent secretion of transgene products into the circulation. AAV‐RRP‐pbFn‐Fc infection modestly induced Ki67 expression from the crypt extending toward the bottom region of Villi (Figure 3F). Hence, our results confirm that RRP‐pbFn activates Wnt/β‐catenin signaling in vivo, altering metabolic liver zonation and inducing intestinal villi proliferation.

### 2.5 RRP‐pbFn Does Not Bind to LRP, but Triggers FZD‐LRP Interaction for Wnt/β‐Catenin Signaling Activation

**Figure 4:**
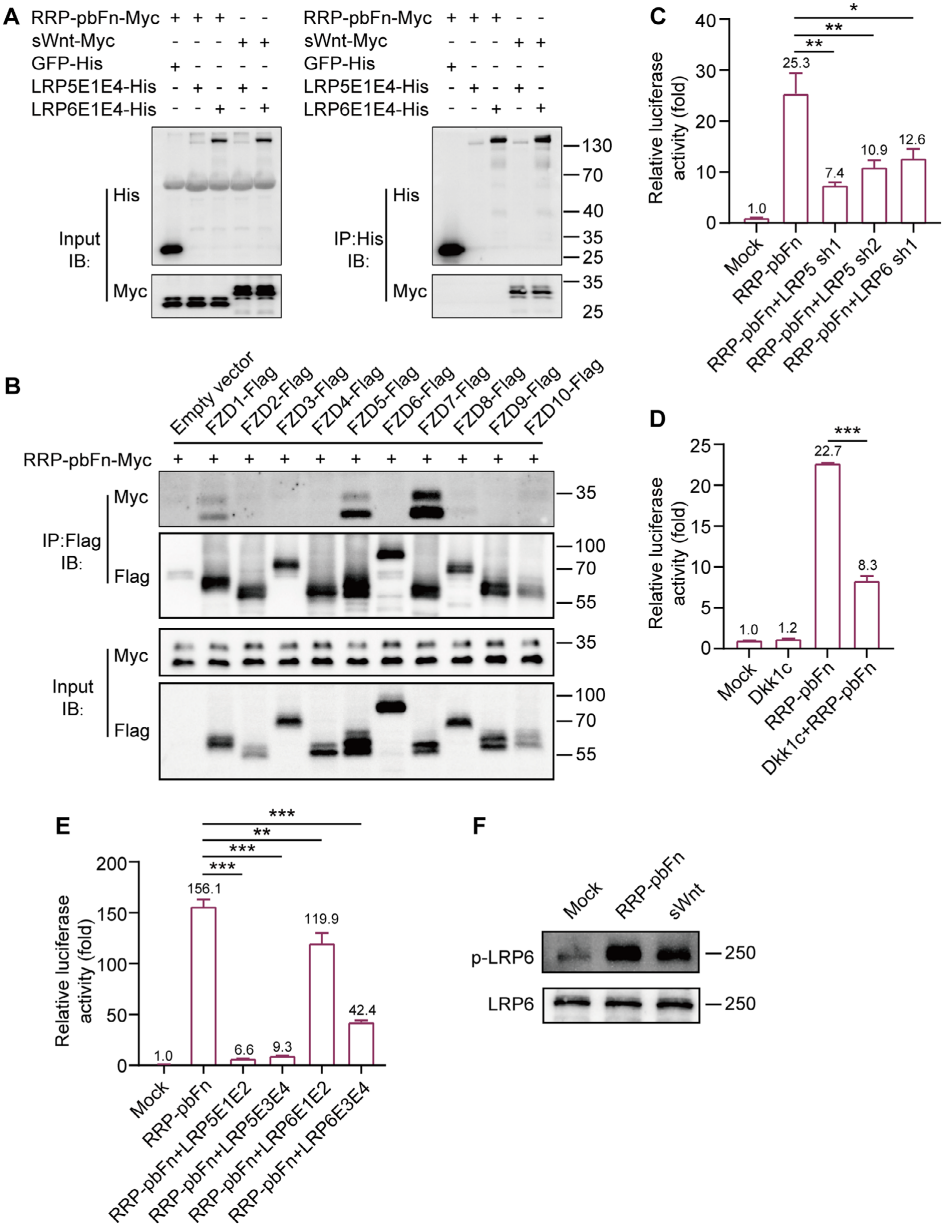
RRP‐pbFn does not bind to LRP, but triggers FZD‐LRP interaction for Wnt/β‐catenin signaling activation. A) Co‐IP analysis of RRP‐pbFn and LRP5/6 ectodomain. Supernatant was subjected to immunoprecipitation of His‐tagged proteins followed by immunoblotting for anti‐Myc. B) Co‐IP analysis of the interaction between RRP‐pbFn and FZDs. Assays were performed in HEK 293T cells transfected with FZDs‐Flag, medium was replaced with RRP‐pbFn‐Myc conditioned medium 12 h post‐transfection, and Flag‐tagged proteins were immunoprecipitation with Flag‐beads. C) TOP‐Flash luciferase reporter assay showing the Wnt activity induced by overexpression of RRP‐pbFn with LRP5 sh, LRP6 sh. D) TOP‐Flash luciferase reporter assay showing the Wnt activity induced by overexpression of Dkk1c, RRP‐pbFn, or their combination. E) TOP‐Flash luciferase reporter assay showing the impact of L5E1E2, L5E3E4, L6E1E2, or L6E3E4 on RRP‐pbFn‐mediated activation of Wnt/β‐catenin signaling. TOP‐Flash data represent mean ± SD; *n* = 3 independent experiments. *p < 0.05; **p < 0.01; ***p < 0.001. F) Western blot analysis of p‐LRP6 induced by RRP‐pbFn and sWnt.

The activation of Wnt/β‐catenin signaling occurs when the Wnt ligand binds to the coreceptors FZD and LRP5/6, leading to the dimerization of two receptors.^[^ ^1^ ^]^ It was known that Wnt/β‐catenin signaling activation by RRP‐pbFn was dependent on the recruitment of FZD receptor. In order to determine whether RRP‐pbFn activated Wnt/β‐catenin signaling through binding directly to the LRP5/6 receptor and the selectivity for FZDs, we conducted co‐immunoprecipitation (Co‐IP) in HEK 293T cells. The outcomes revealed that LRP5 ectodomain (LRP5E1E4) and LRP6 ectodomain (LRP6E1E4) Co‐IP with sWnt, but not with RRP‐pbFn (**Figure** **4**A), and RRP‐pbFn strongly interacted with FZD5 and FZD7, and modestly interacted with FZD1 (Figure 4B), suggesting that the activation of Wnt/β‐catenin signaling by RRP‐pbFn targets individual type of cell‐surface receptors (FZD) without binding to the LRP5/6 receptor.

To determine whether RRP‐pbFn‐activated FZD could stimulate Wnt/β‐catenin signaling in absence of LRP, short hairpin RNAs (shRNAs) were utilized to suppress the expression of LRP5/6, and knockdown efficiency was confirmed by qRT‐PCR analysis (Figure S6A,B, Supporting Information). It was found that LRP5/6 shRNAs mediated knockdown of LRP5 and LRP6 inhibited RRP‐pbFn or sWnt‐induced Wnt responses demonstrated by the reduced expression of the TOP‐Flash reporter in HEK 293T cells (Figure 4C; Figure S6C, Supporting Information). Then, we overexpressed Dkk1c to restrain the function of LRP5/6 in the initiation of Wnt signal transduction, which resulted in abolishing Wnt/β‐catenin signaling activation by sWnt and inhibiting RRP‐pbFn‐induced Wnt/β‐catenin signaling activation (Figure 4D; Figure S6D, Supporting Information). To further explore the impact of interfering with endogenous transmembrane receptors FZD and LRP5/6 dimerization in Wnt/β‐catenin signaling activation, we overexpressed LRP5 ectodomain LRP5E1E2, LRP5E3E4, and LRP6 ectodomain LRP6E1E2, LRP6E3E4. We found that weakening of the interaction between endogenous receptors FZD and LRP5/6 restrained RRP‐pbFn or sWnt‐induced Wnt/β‐catenin signaling activation (Figure 4E; Figure S6E, Supporting Information). Phosphorylation of the cytoplasmic tail of LRP6 is crucial to β‐catenin stabilization and subsequent Wnt/β‐catenin activation.^[^ ^1^ ^]^ Indeed, RRP‐pbFn induced phosphorylation of LRP6, which was more intensive than that of sWnt (Figure 4F). These findings indicate that RRP‐pbFn activated Wnt/β‐catenin signaling without binding to the LRP5/6 receptor, but rather triggers FZD‐LRP interaction.

Replacing Ile52 and Met55 of VVD with positively charged arginine residues (I52R/M55R) created pMag, while using negatively charged aspartic acid and glycine (I52D/M55G) generated nMag.^[^ ^26^ ^]^ We found pMag‐pbF significantly activated Wnt/β‐catenin signaling, but nMag‐pbF or VVD‐pbF failed to do so (Figure 1E). Notably, substituting positively charged arginine residue R52 with I (RRP(R52I)‐pbFn) abolished its Wnt activation capacity (Figure 1K). To assess if the charge of amino acid affects RRP‐pbFn's ability to activate Wnt/β‐catenin signaling, we substituted R52 with other positively charged lysine or histidine residues. Unexpectedly, both RRP(R52K)‐pbFn and RRP(R52H)‐pbFn showed no activity, yet they still interacted with FZD7, demonstrating that the Wnt activation mechanism of pMag‐pbF was charge independent (Figure S7A,B, Supporting Information). To investigate whether RRP mimics R‐spondin in inducing ZNRF3 inhibition and increasing the membrane level of Wnt receptors,^[^ ^21^ ^]^ we evaluated the protein levels of FZD7 of cells treated with these ligands. Unlike R‐spondin1, which increased FZD7, RRP‐pbFn or sWnt did not alter the FZD7 protein level (Figure S7C, Supporting Information). We examined additional signaling pathways Smo, TGF‐β and Yap, which were indirectly linked to the Wnt pathway to determine whether pMag or RRP‐pbFn activated these pathways, which ultimately converged on Wnt/β‐catenin signaling. The results indicated that neither pMag, pMag‐pbFn, nor RRP‐pbFn activated these pathway (Figure S7D,E, Supporting Information).

### 2.6 Generation of High‐Efficiency LRP Antagonist by Fusing RRP with Dkk1c

**Figure 5:**
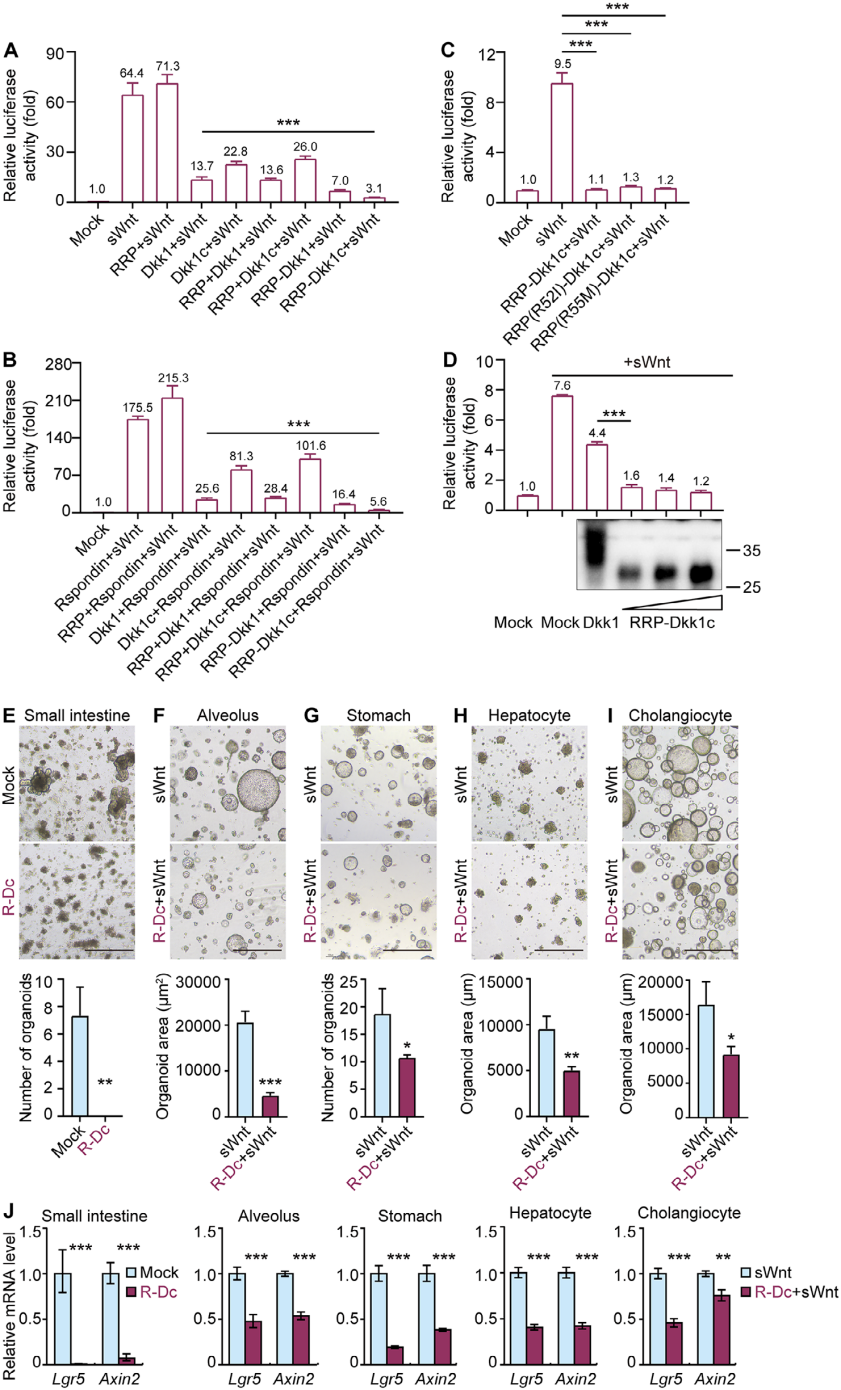
Synthetic LRP antagonist RRP‐Dkk1c is a more potent Wnt/β‐catenin signaling inhibitor. A,B) TOP‐Flash luciferase reporter assay for evaluating Wnt activity in HEK 293T cells induced by 50 ng mL^−1^ sWnt (A) or 50 ng mL^−1^ sWnt+250 ng mL^−1^ R‐spondin1 (B), and in combination with overexpressing the indicated proteins. C) TOP‐Flash luciferase reporter assay for evaluating Wnt activity in HEK 293T cells induced by 50 ng mL^−1^ sWnt, and in combination with overexpressing RRP‐Dkk1c, RRP(R52I)‐Dkk1c, or RRP(R55M)‐Dkk1c. D) TOP‐Flash luciferase reporter assay for evaluating Wnt activity induced by 50 ng mL^−1^ sWnt, and in combination with overexpressing RRP‐Dkk1c or Dkk1. The bottom panel is detection of Dkk1 and RRP‐Dkk1c in supernatant after overexpression without sWnt by Western blot for anti‐Dkk1. E) Representative bright‐field images of mouse small intestinal organoids expanded in culture media with or without 500 ng mL^−1^ recombinant RRP‐Dkk1c. The bottom panel is the quantification of the organoid number. R‐Dc: RRP‐Dkk1c. F‐I) Representative bright‐field images of mouse organoids derived from alveolus (F), stomach (G), and hepatocyte (H) expanded in culture media with 50 ng mL^−1^ sWnt, and human cholangiocyte (I) expanded in culture media containing 50 ng mL^−1^ sWnt and 3 µM IWP‐2, and supplemented with or without 500 ng mL^−1^ recombinant RRP‐Dkk1c. The bottom panels are quantification of the organoid number (stomach) or organoid area (alveolus, hepatocyte, and cholangiocyte). The scale bar represents 500 µm. R‐Dc: RRP‐Dkk1c. J) qRT‐PCR analysis of gene expression of Wnt/β‐catenin signaling target genes. R‐Dc: RRP‐Dkk1c. Data represent mean ± SD, and *n* = 3 for each organoid type. *p < 0.05; **p < 0.01; ***p < 0.001.

Given that RRP(R52I)‐pbFn inhibited the Wnt/β‐catenin signaling activation induced by RRP‐pbFn (Figure S3B, Supporting Information), we proceeded to identify its potential as an antagonist against sWnt‐induced Wnt/β‐catenin signaling, revealing a modest antagonistic effect (Figure S8, Supporting Information). To develop a synthetic antagonist for effectively blocking Wnt ligand or sWnt‐dependent Wnt/β‐catenin signaling activation, we replaced pbFn with the high‐affinity LRP5/6 receptor binder Dkk1 or Dkk1c, considering the partial redundancy of 10 FZD receptors and the binding specificity of pbF to FZD1, 2, 5, 7, and 8.^[^ ^28^, ^37^ ^]^ As expected, both Dkk1 and Dkk1c partially blocked sWnt‐induced Wnt responses with or without R‐spondin1, while RRP‐Dkk1 exhibited further inhibition and RRP‐Dkk1c fully suppressed Wnt/β‐catenin signaling activation to less than 5% (**Figure** **5**A,B), with RRP‐Dkk1c demonstrating superior inhibitory activity compared to RRP(R52I)‐pbFn (Figure S8, Supporting Information). Moreover, we confirmed RRP‐Dkk1c interacted with LRP6 ectodomain (Figure S9, Supporting Information). Through mutagenesis of RRP(R52I) and RRP(R55M) (Figure 1K), we unexpectedly observed that RRP(R52I)‐Dkk1c and RRP(R55M)‐Dkk1c inhibited Wnt/β‐catenin signaling activation by sWnt (Figure 5C). These results suggest that RRP played distinct regulatory roles in fusion with pbFn and Dkk1c.

### 2.7 RRP‐Dkk1c Shows a Much Higher Capacity for Wnt/β‐Catenin Signaling Inhibition than Currently Available LRP Antagonist

We compared the capacity of Dkk1 with RRP‐Dkk1c for Wnt/β‐catenin signaling inhibition in a dose‐dependent manner using Wnt reporter assays in HEK 293T cells. RRP‐Dkk1c showed higher capacity than Dkk1 for inhibiting Wnt/β‐catenin signaling activation induced by sWnt (Figure 5D). Additionally, RRP‐Dkk1c exhibited a smaller molecular weight compared to Dkk1 (Figure 5D). In conclusion, water‐soluble LRP antagonist RRP‐Dkk1c exhibited a smaller molecular weight but more potent inhibitory activity than Dkk1.

We then evaluated the function of LRP antagonist RRP‐Dkk1c during the growth of organoids. Treatment of mouse intestinal crypt with recombinant RRP‐Dkk1c led to a notable reduction in overall organoid viability (Figure 5E). In addition, the application of recombinant RRP‐Dkk1c resulted in a decreased number of mouse stomach‐derived organoids and the size of alveolus‐, hepatocyte‐, and human cholangiocyte‐derived organoids cultured in sWnt‐supplemented medium (Figure 5F–I). Consistently, the mRNA levels of Wnt target genes *Lgr5* and *Axin2* were significantly down‐regulated by recombinant RRP‐Dkk1c in these organoids (Figure 5J). In conclusion, our findings provide evidence of the potent inhibitory effect of synthetic LRP antagonist RRP‐Dkk1c in impeding organoid growths.

### 2.8 RRP‐Dkk1c Abolishes the Formation of CT26‐Derived Colon Cancer Xenograft

**Figure 6:**
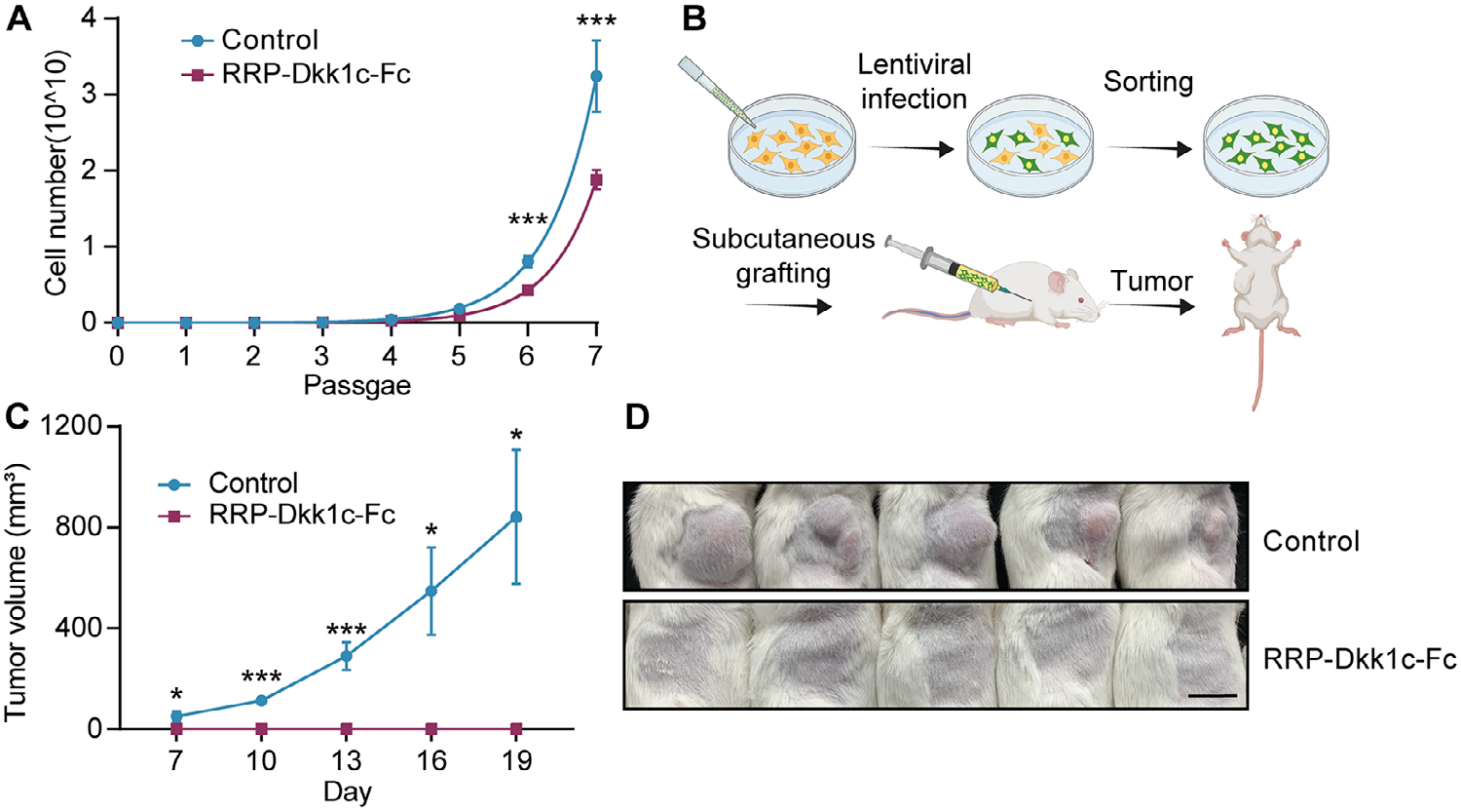
RRP‐Dkk1c abolishes the formation of CT26‐derived colon cancer xenograft. A) Growth curve of stable CT26 cells expressing RRP‐Dkk1c‐Fc or Fc (control) from Passage 1 to Passage 7 from three independent experiments using stable CT26 cell line. B) A scheme depicting the subcutaneous injection of CT26 for the xenograft model. The mice were subcutaneously injected with 5.0 × 10^5^ sorted CT26 cells. C) The volume of subcutaneous xenograft tumors (*n*  =  5 mice per group). D) Photograph of tumors from mice injected with control cells or stable CT26 cells expressing RRP‐Dkk1c‐Fc on day 19. Data represent mean ± SD, and *n* = 5 mice per group. *p < 0.05; **p < 0.01; ***p < 0.001.

Kaplan‐Meier analysis of 595 colorectal cancer patients from the Human Protein Atlas Dataset revealed that high Dkk1 expression in colon cancer samples (*n* = 455) was significantly associated with increased survival rates compared to samples with low Dkk1 expression (*n* = 140) (Figure S10, Supporting Information), suggesting the inhibitory role of Dkk1 in Wnt/β‐catenin signaling is correlated with higher overall survival rate in colorectal cancer patients. To evaluate the potential of RRP‐Dkk1c as a tumor growth inhibitor in vivo, a xenograft model was established by subcutaneously injecting mice with stable CT26 cells, which enable rapid proliferation in vitro (**Figure** **6**A,B). CT26, an undifferentiated colon carcinoma cell line lacking the Apc mutation,^[^ ^38^ ^]^ was selected as an appropriate model for investigating the effects of RRP‐Dkk1c. Lentivirus‐infected cells were sorted to generate stable CT26 cells expressing RRP‐Dkk1c‐Fc or Fc as a control. Notably, the colony formation assay revealed a significant suppression of colony growth in RRP‐Dkk1c‐Fc stable CT26 cells, with a cell count of 1.88 × 10^10^ compared to 3.24 × 10^10^ in control cells after the seventh passage (Figure 6A). In agreement with in vitro findings, injection of 5.0 × 10^5^ control cells into mice resulted in tumor formation, whereas mice injected with RRP‐Dkk1c‐Fc stable CT26 cells showed no signs of tumor mass (Figure 6C,D). These results collectively demonstrate the potent tumor‐suppressive properties of RRP‐Dkk1c in colon cancer.

## 3 Discussion

In summary, we describe the Wnt/β‐catenin signaling agonist/antagonist targeting FZD/LRP receptors. Synthetic RRP‐pbFn serves as a water‐soluble FZD agonist, overcoming the limitations of natural ligands including high hydrophobicity and low activity. This advancement opens avenues for organoid expansion and the development of Wnt‐dependent regenerative medicine. On the contrary, synthetic LRP antagonist RRP‐Dkk1c potently inhibits Wnt/β‐catenin signaling for cancer therapeutics. Collectively, these modulators hold immense potential for scientific research and a wide range of biomedical applications.

The hydrophobicity of WNTs has impeded their derivatives for research and therapeutics. The identification of surrogate Wnt (pbF‐Dkk1c) constitutes a remarkable stride in the advancement of research tools.^[^ ^16^ ^]^ Further, the improved surrogate Wnt (Dkk1c‐pbF) exhibits sustained efficacy in activating Wnt/β‐catenin signaling.^[^ ^17^ ^]^ In spite of these advancements, we report a more powerful Wnt agonist RRP‐pbFn which consisted of the first 120 aa of optogenetic tool pMag with the first 130 aa of pbF. Moreover, the water‐soluble RRP‐pbFn, characterized by its lower molecular weight, overcomes the challenges associated with the large molecular size of antibody‐based Wnt agonists, which comprise two binding sites for FZD and LRP.^[^ ^17^, ^18^, ^19^ ^]^ Endowed with those merits, RRP‐pbFn boosts the growth of various mouse and human organoids, indicating that RRP‐pbFn is a promising substitute for canonical Wnt agonists (Figure 2), offering new standardized protocols for organoid cultivation. In addition, utilizing AAV delivery system combined with R‐spondin1 in vivo, RRP‐pbFn demonstrates potent regenerative effects on liver and intestine (Figure 3). The activity of RRP‐pbFn in vivo opens up novel therapeutic opportunities for the stem cell‐based tissue regeneration.^[^ ^39^, ^40^ ^]^

At the cell surface, the Wnt/β‐catenin signaling axis involves a ternary complex composed of the Wnt ligand, FZD receptor, and LRP receptor.^[^ ^41^ ^]^ The fusion protein RRP‐pbFn, combining RRP with a binder targeting FZD receptors, robustly stimulates Wnt/β‐catenin signaling (Figure 1). Conversely, RRP‐Dkk1c, fusing RRP with a binder for LRP receptors, exhibits enhanced inhibitory potency compared to Dkk1c alone (Figure 5A,B). These data collectively underlie a potential preference of RRP toward LRP receptors. RRP‐pbFn does not directly bind to LRP but instead facilitates the phosphorylation of LRP6 to activate Wnt/β‐catenin signaling. Additionally, we confirm that this activation is charge‐independent. Since RRP originates from the optogenetic tool pMag/VVD, where VVD can form a dimer, it remains unclear whether RRP‐pbFn itself forms dimers or potentially large complexes through liquid‐liquid phase separation to activate the Wnt/β‐catenin signaling. Further investigation is needed to elucidate the molecular mechanisms and the structure‐function relationship involved in the phosphorylation of LRP6 following RRP‐pbFn binding to FZD, as well as the role of RRP in the activation of Wnt/β‐catenin signaling. To fully understand how RRP‐pbFn activates Wnt/β‐catenin signaling, additional studies like mass spectrometry, proximity labeling techniques such as TurboID, and structural biology approaches are required. We anticipate that these efforts will provide deeper insights into the underlying mechanisms.

Tumor cell proliferation can differ between in vitro and in vivo setups, since the latter involves more complicated niche factors. However, our in vitro experiments cleanly demonstrate that RRP‐Dkk1c‐Fc‐mediated proliferation inhibition is tumor cell‐intrinsic and dependent on Wnt/β‐catenin signaling (Figure 6A). Further, in vivo transplantation assay also supported the conclusion. Actually, the inhibitory effect of RRP‐Dkk1c‐Fc is more powerful than in vitro settings (the tumor is nearly disappearing, Figure 6C,D), suggesting the RRP‐Dkk1c‐Fc not only affecting cell proliferation intrinsically, but also suppressing tumor formation through disturbing its niche. The development and progression of various human cancer types have been associated with enhanced expression of Wnt receptors, attributed to the mutational inactivation of the ubiquitin ligases RNF43/ZNRF3. This underscores the potential of RRP‐Dkk1c as a therapeutic strategy for targeting Wnt‐dependent cancers. The varied tertiary structures of protein‐derived agonists and antagonists augment their interactions with specific receptor pockets, granting them with heightened potency and diminished toxicity compared to small molecules.^[^ ^42^ ^]^

Our research provides an important research paradigm for the exploration of key receptor modulators targeting FZD/LRP receptors, promoting the translational application of signal transduction. Moreover, the designed synthetic protein arsenal through artificial or artificial intelligence‐based design opens up new prospects for stem cell research, organoid development, regenerative medicine, and disease therapy.

## 4 Experimental Section

### Animals and Human Biopsies

BALB/c mice aged 6–8 weeks were purchased from GemPharmatech. All animal experiments were performed in accordance with protocols (#IDM2024020) approved by the Institutional Animal Care and Use Committee at Institute of Developmental Biology and Molecular Medicine of Fudan University.

The research was conducted in accordance with the Declaration of Helsinki, and the use of human tissues for this study was approved by the Institutional Ethical Committee of Obstetrics and Gynecology Hospital of Fudan University and the approval number was 2018–77. Written informed consent was obtained from all participants prior to their involvement in the study.

### Plasmids

All constructs, except where indicated, were cloned into pcDNA3.1(+) Myc vector (Thermo Fisher) with an N‐terminal tissue plasminogen activator (tPA) signal sequence (MDAMKRGLCCVLLLCGAVFVSA) for overexpression. The sequence of the Vantictumab was retrieved from the published patent, which was subsequently reformatted into pbF. The cDNA of pMag, pbF, and Dkk1c (the C‐terminal domain of human Dkk1, residues 177–266) were synthesized by Genscript. The plasmid constructs pMag‐pbF and RRP‐Dkk1c, designed for secretion of the encoded protein, were generated by cloning pMag or RRP, along with a flexible linker peptide GSGSG, followed by pbF or Dkk1c, respectively. Plasmids were generated for Co‐IP experiment, including constructs encoding RRP‐pbFn and sWnt with a C‐terminal Myc‐tag, as well as constructs encoding LRP5E1E4, LRP6E1E4, and GFP with a C‐terminal 6x His‐tag. RRP‐Dkk1c with a C‐terminal Fc‐tag was cloned into the lentiviral pLVX‐CMV‐puro‐GFP plasmid. FZD1‐10‐Flag and LRP6ECD‐HA were gifts from Maorong Cheng lab, Yunnan university. All short hairpin RNA (shRNA) sequences were cloned into the pLKO.1‐Puro vector and the sequences are presented in Table S1 (Supporting Information).

### Conditioned Media and Protein Purification

HEK 293T and CT26 cells were cultured in DMEM (Gibco) supplemented with 10% FBS (Gibco) and 1% Penicillin‐Streptomycin (Gibco) at 37 °C in a 5% CO2 environment. The conditioned media was prepared through transient transfection of 293T cells. The medium was replaced with fresh medium 12 h post‐transfection and cells were further incubated for 24 h. Subsequently, cells were removed by centrifugation. Briefly, for the production of RRP‐pbFn and RRP‐Dkk1c with a C‐terminal Fc‐tag recombinant protein, HEK 293T cells were transiently transfected and expression was proceeded for 5 days at 37 °C and 8% CO2 with shaking at 125 rpm. After expression, cells were removed by centrifugation and proteins were purified from the conditioned media using rProtein A Sepharose (GE healthcare). The purified proteins were buffer‐exchanged into a formulation buffer (20 mM His‐HAc, 150 mM NaCl, pH 5.5). Recombinant Fc‐tagged RRP‐pbFn and Fc‐tagged RRP‐Dkk1c were abbreviated as recombinant RRP‐pbFn and RRP‐Dkk1c, respectively.

### TOP‐Flash Reporter Assay

HEK 293T cells were plated into a 48‐well cell culture plate (Thermo) and cultured overnight to reach 60–80% confluency. 293T cells were transfected with 50 ng of TOP‐Flash plasmid encoding the firefly luciferase and 10 ng of *Renilla* luciferase plasmid per well using VigoFect (Vigorous) according to the manufacturer's instructions. Twelve hours post‐transfection, the medium was changed to fresh medium supplemented with 250 ng mL^−1^ R‐spondin1, 50 ng mL^−1^ sWnt, 50% pMag‐pbFn conditioned media, or left untreated for 24 h as indicated. Cells were lysed with Luciferase Cell Culture Lysis Reagent (Promega) and luciferase activity was measured according to the manufacturer's protocols. The ratios of firefly luminescence to *Renilla* luminescence were calculated and normalized to the control samples that were left untreated. In terms of illumination, 24 h after transfection, the cells were illuminated with blue light (470 ± 20 nm, 1.0 mW cm^−2^, 30 s pulse every 3 min) for 24 h or kept in darkness.

### Small Intestinal Organoids

Jejunal tissue (≈10 cm) was isolated, longitudinally cut, and washed with cold DPBS (Thermo). Subsequently, the villi were carefully scraped off, and tissue was minced and incubated in DPBS containing 5 mM EDTA (Gibco) for 30 min at 4 °C. After transferring the tissue into a tube with fresh DPBS, crypts were released by vigorously shaking then passed through a 70 µm cell strainer (BD). The isolated crypts were pelleted at 600 rpm for 5 min, resuspended in 25 µL Matrigel (bioGenous), and seeded in a 24‐well plate. After polymerization at 37 °C for 15 min, culture medium consisting of Advanced DMEM/F12 (Thermo) supplemented with G27 supplement (bioGenous), N‐Acetylcysteine (Sigma‐Aldrich), GlutaMAX (Gibco), 1% Penicillin‐Streptomycin (Invitrogen), 500 ng mL^−1^ R‐spondin1 (bioGenous), 100 ng mL^−1^ Noggin (bioGenous) and 50 ng mL^−1^ EGF (bioGenous) in the absence or presence of 200 ng mL^−1^ RRP‐pbFn or 500 ng mL^−1^ RRP‐Dkk1c was added to each well. Images of the 3D cultured organoids were acquired on Day 5. For organoid imaging, images were captured from a plane that includes as many organoids as possible to accurately reflect their growth status using a low‐magnification lens with a large depth of field.

### Alveolus Organoids

After rinsing the dissected mouse lung with DPBS, incision was made along the edge of the lung lobe. The piece of tissue was subjected to a freshly prepared digestion solution consisting of Advanced DMEM/F12 supplemented with 10 µM Y‐27632 (Sigma‐Aldrich), 400 U mL^−1^ collagenase NB 4 (Nordmark), and 10 U mL^−1^ Dnase I (Roche) for 50 min at 37 °C. The digestion solution was pipetted up and down and then filtered through a 70 µm cell strainer. After centrifugation of the filtrate at 350 g for 3 min, the supernatant was discarded, and Red Blood Cell Lysis Buffer (eBioscience) was added for 3 min followed by DPBS washing. The mixture was centrifuged and then embedded in Matrigel. The culture medium consisted of Advanced DMEM/F12 supplemented with G27, GlutaMAX, N‐Acetylcysteine, 1% Penicillin‐Streptomycin, 10 mM HEPES (Gibco), 5 mM Nicotinamide (Sigma‐Aldrich), 0.5 µM A83‐01 (Tocris), 10 µM Y‐27632, 0.5 µM SB‐202190 (Sigma‐Aldrich), 500 ng mL^−1^ R‐spondin1, 100 ng mL^−1^ Noggin, 50 ng mL^−1^ EGF, 100 ng mL^−1^ FGF10 (bioGenous) and 25 ng mL^−1^ FGF7 (bioGenous) in the absence or presence of 50 ng mL^−1^ sWnt, 200 ng mL^−1^ RRP‐pbFn or 500 ng mL^−1^ RRP‐Dkk1c. Images of the 3D cultured organoids were acquired on Day 5.

### Gastric Organoids

Gastric glands units were isolated from mouse pyloric stomach as previously described with some modifications.^[^ ^43^ ^]^ Briefly, the stomach was opened along the greater curvature, and the pylorus was isolated followed by DPBS washing. The muscular layer of the stomach was removed and the remaining epithelia was divided into 5 mm pieces and incubated in digestion solution consisting of Advanced DMEM/F12 supplemented with 2 mg mL^−1^ collagenase type I (Thermo), 100 µg mL^−1^ primocin (Invivogen), 10% FBS, and 10 U mL^−1^ DNase I for 20–30 min at 37 °C. The isolated gastric glands were filtered through a 70 µm cell strainer then a total of 200 glands mixed with Matrigel were plated in 24‐well plate. Gastric culture medium consisted of Advanced DMEM/F12 supplemented with G27, GlutaMAX, N‐Acetylcysteine, 1% Penicillin‐Streptomycin, Y‐27632, 500 ng mL^−1^ R‐spondin1, 100 ng mL^−1^ Noggin, 50 ng mL^−1^ EGF, 100 ng mL^−1^ FGF10, 10 nM Gastrin I (Sigma‐Aldrich), and 100 µg mL^−1^ Primocin in the absence or presence of 50 ng mL^−1^ sWnt, 200 ng mL^−1^ RRP‐pbFn or 500 ng mL^−1^ RRP‐Dkk1c. Images of the 3D cultured organoids were acquired on Day 4.

### Hepatocyte Organoids

Primary hepatocytes were isolated from mice as previously described.^[^ ^44^ ^]^ Briefly, after placing catheter into the portal vein, the inferior vena cava was cut and the liver was perfused with pre‐warmed perfusion medium. Then, perfusion was performed with pre‐warmed digestion medium including collagenase type IV and Ca^2+ ^for 3–5 min. After dissociation, cells were filtered through a 70 µm cell strainer. Hepatocytes were further separated and purified by centrifugation at 50 g for 3 min and percoll gradient centrifugation was performed. In a 24‐well plate, 5 × 10^4^ isolated hepatocytes mixed with Matrigel at a concentration of 75% diluted by culture medium were used per well. Culture medium was composed of Advanced DMEM/F12 supplemented with G27, GlutaMAX, N‐Acetylcysteine, 1% Penicillin‐Streptomycin, HEPES, Nicotinamide, 1 µM A83‐01, Y‐27632, 500 ng mL^−1^ R‐spondin1, 50 ng mL^−1^ EGF, 100 ng mL^−1^ FGF10, 10 nM Gastrin I, 25 ng/ mL HGF (bioGenous) and 100 ng mL^−1^ TGF‐α (R&D Systems) in the absence or presence of 50 ng mL^−1^ sWnt, 200 ng mL^−1^ RRP‐pbFn or 500 ng mL^−1^ RRP‐Dkk1c. Images of the 3D cultured organoids were acquired on Day 4.

### Human Cholangiocyte Organoids

Cholangiocyte organoids from donor livers were generated as previously described.^[^ ^45^ ^]^ Briefly, livers were minced and digested by incubation in advanced DMEM/F12 supplemented with 2 mg mL^−1^ collagenase type I and 10 U mL^−1^ DNase I for 20–30 min at 37 °C. Digestion solution was diluted by adding cold Advanced DMEM/F12 supplemented with 1% Penicillin/Streptomycin and 1% FBS. The cell suspension was filtered through a 70 µm cell strainer and centrifuged at 400 g, 4 °C for 3 min. Supernatant was removed, and the cell pellet was suspended with Matrigel which was allowed to solidify for 15 min at 37 °C before cholangiocyte organoid culture medium was added. Culture medium was composed of Advanced DMEM/F12 supplemented with G27, GlutaMAX, N‐Acetylcysteine, Penicillin‐Streptomycin, HEPES, Nicotinamide, 2 µM A83‐01, 500 ng mL^−1^ R‐spondin1, 50 ng mL^−1^ EGF, 100 ng mL^−1^ FGF10, 10 nM Gastrin I, 25 ng mL^−1^ HGF, and 10 µM Forskolin (Sigma‐Aldrich). After passaging cholangiocyte organoids, 3 µM IWP‐2 was added in culture medium to inhibit endogenous Wnt lipidation and secretion in the absence or presence of 50 ng mL^−1^ sWnt, 200 ng mL^−1^ RRP‐pbFn or 500 ng mL^−1^ RRP‐Dkk1c. Images of the 3D cultured organoids were acquired on Day 5.

### Immunoprecipitation and Immunoblotting

Secreted RRP‐pbFn‐Myc, sWnt‐Myc, GFP‐His, LRP5E1E4‐His, and LRP6E1E4‐His were overexpressed in HEK 293T cells. After expression, cells were removed by centrifugation and supernatant was mixed with 1:1 as indicated, then HEK 293T cells were overlaid with the mixture for 60 min at 37 °C. The mixture was used for immunoprecipitation with Ni Sepharose excel (GE healthcare) according to the manufacturer's protocols. Immunoblotting was performed using the following antibodies: α‐His (Proteintech, 1:3000), α‐Myc (Abclonal, 1:5000), α‐HA (Abmart, 1:1000), α‐FZD7 (HUABIO, 1:1000), α‐GAPDH (Proteintech, 1: 5000), α‐Flag (Sigma, 1:1000), IPKine HRP Goat Anti‐Rabbit IgG HCS (Abbkine, 1:2500) and IPKine HRP Goat Anti‐Mouse IgG LCS (Abbkine, 1:2500). The membrane proteins of HEK 293T cells were isolated using membrane protein extraction kit (BestBio, Shanghai).

### Immunofluorescence

The small intestine and liver of mouse were fixed overnight in 4% paraformaldehyde at 4 °C and processed for paraffin embedding. Subsequently, 5‐µm sections were stained with the following antibodies following citrate antigen retrieval and blocking with 10% normal donkey serum: mouse anti‐glutamine synthetase (BD Bioscience, 1:2000), mouse anti‐Ki67 (BD, 1:500). Then sections were incubated with Alexa Fluor 488 (Jackson ImmunoResearch, 1:200) and Cy3 (Jackson ImmunoResearch, 1:200)‐labeled secondary antibodies, counterstained with DAPI at room temperature in the dark. Images were captured with Olympus FV3000 Confocal Laser Scanning Microscope.

### RNA Extraction and Quantitative Real‐Time PCR

Total RNA was extracted using RNAprep Pure Micro Kit (Tiangen Biotech) according to manufacturer's protocols. Purified RNA was reverse‐transcribed into cDNAs with GoScript Reverse Transcription System (Promega) according to the manufacturer's instruction. Quantitative Real‐Time PCR was performed using SYBR Green PCR Master Mix (Bimake) and detected by CFX384 Touch System (Bio‐rad). Expression levels were normalized to the reference gene *Gapdh*, and the primers were listed in Table S2 (Supporting Information).

### In Vivo Experiments

A single intravenous injection of 3.0 × 10^11^ vg per mouse AAV‐8 expressing Fc (control) or RRP‐pbFn‐Fc was administered. After 10 days injection of AAV‐8, mice were injected intravenously with 200 µg per mouse recombinant R‐spondin1 or PBS daily for 6 days. Intestinal tissues and livers were collected 24 h after the last R‐spondin1 injection (*n* = 3 mice per group).

### Mouse Xenograft Model

CT26 tumor cells infected with either RRP‐Dkk1c‐Fc or Fc (control) lentivirus were sorted for GFP expression, 5.0 × 10^5^ sorted GFP^+^ cells were suspended in 125 µL phosphate buffered saline and subcutaneously injected into the right flanks of BALB/c mice. Tumor growth was measured with vernier calipers and tumor volumes were calculated using the modified ellipsoid formula (0.5 × length × width × width).

### Colony Formation Assay

1.0 × 10^6^ sorted GFP^+^ CT26 tumor cells were seeded per 6‐well plate and cultured in medium until they reached 80–90% confluency. CT26 were then dissociated into single cells using 0.25% Trypsin and the number of cells was counted. The counted cells were considered as passage 1, and 1.0 × 10^6^ cells were reseeded per 6‐well plate for subsequent passages. This process was repeated for a total of seven passages, and each experiment was performed with 5 replicates.

### Survival Analysis

The clinical data was obtained from Human Protein Atlas Dataset, and a total of 595 colorectal cancer patients were included for survival analysis. Dkk1 expression levels were determined using FPKM (fragments per kilobase of transcript per million fragments mapped) values, with FPKM > 0 considered as high Dkk1 expression and FPKM = 0 considered as low Dkk1 expression.

### Protein Structure Modeling

The protein sequences in FASTA format were supplied for SWISS‐MODEL.^[^ ^46^ ^]^ Subsequently, 3hjk.1 was utilized to identify the optimal template for protein modeling. Through aligning the target sequence with the template, conserved sequences were identified. Based on the homology information, full‐atom protein models were generated. The visualization settings of the model were adjusted using ChimeraX.

### Quantification and Statistical Analysis

Comparisons of the rings of GS^+^ hepatocytes and the number of villi were performed in a double‐blinded manner. A minimum of 3 images were analyzed per animal. Statistical analyses were conducted using GraphPad Prism 8. For comparisons between two groups, an unpaired two‐tailed Student's t‐test was used. For comparisons involving more than two groups, a one‐way ANOVA was applied to determine statistical significance. A log‐rank test was used for survival analysis. The mean and standard deviation (SD) or standard error (SE) are reported in the figure legends. A p‐value < 0.05 was considered statistically significant. All sample sizes (n), listed in the figure legends, represent biological replicates.

## Conflict of Interest

The authors declare no conflict of interest.

## Author Contributions

Q.D., C.N., and B.Z. designed the experiments; Q.D., J.W., Z.L., D.Y., H.Y., J.W., X.L., and H.H. performed the experiments; Q.D., C.N., and B.Z. analyzed the data; B.Z. supervised the work; Q.D. and B.Z. wrote the paper.

## Supporting information



Supporting Information

## Data Availability

The data that support the findings of this study are available from the corresponding author upon reasonable request.

## References

[1] R. Nusse , H. Clevers , Cell 2017, 169, 985. 28575679 10.1016/j.cell.2017.05.016

[2] C. Niehrs , Nat. Rev. Mol. Cell Biol. 2012, 13, 767. 23151663 10.1038/nrm3470

[3] H. Clevers , R. Nusse , Cell 2012, 149, 1192. 22682243 10.1016/j.cell.2012.05.012

[4] E. Y. Rim , H. Clevers , R. Nusse , Annu. Rev. Biochem. 2022, 91, 571. 35303793 10.1146/annurev-biochem-040320-103615

[5] H. Clevers , Cell 2006, 127, 469. 17081971 10.1016/j.cell.2006.10.018

[6] M. J. Perugorria , P. Olaizola , I. Labiano , A. Esparza‐Baquer , M. Marzioni , J. J. G. Marin , L. Bujanda , J. M. Banales , Nat. Rev. Gastroenterol. Hepatol. 2019, 16, 121. 30451972 10.1038/s41575-018-0075-9

[7] J. Drost , H. Clevers , Nat. Rev. Cancer 2018, 18, 407. 29692415 10.1038/s41568-018-0007-6

[8] V. Veninga , E. E. Voest , Cancer Cell 2021, 39, 1190. 34416168 10.1016/j.ccell.2021.07.020

[9] B. L. LeSavage , R. A. Suhar , N. Broguiere , M. P. Lutolf , S. C. Heilshorn , Nat. Mater. 2022, 21, 143. 34385685 10.1038/s41563-021-01057-5 PMC12276900

[10] R. S. Jope , G. V. Johnson , Trends Biochem. Sci. 2004, 29, 95. 15102436 10.1016/j.tibs.2003.12.004

[11] L. Wang , N. Wang , W. Zhang , X. Cheng , Z. Yan , G. Shao , X. Wang , R. Wang , C. Fu , Sig. Transduct. Target Ther. 2022, 7, 48. 10.1038/s41392-022-00904-4 PMC8844085 35165272

[12] J. P. Dijksterhuis , B. Baljinnyam , K. Stanger , H. O. Sercan , Y. Ji , O. Andres , J. S. Rubin , R. N. Hannoush , G. Schulte , J. Biol. Chem. 2015, 290, 6789. 25605717 10.1074/jbc.M114.612648 PMC4358105

[13] C. Y. Janda , D. Waghray , A. M. Levin , C. Thomas , K. C. Garcia , Science 2012, 337, 59. 22653731 10.1126/science.1222879 PMC3577348

[14] K. Willert , J. D. Brown , E. Danenberg , A. W. Duncan , I. L. Weissman , T. Reya , J. R. Yates III , R. Nusse , Nature 2003, 423, 448. 12717451 10.1038/nature01611

[15] T. Seino , S. Kawasaki , M. Shimokawa , H. Tamagawa , K. Toshimitsu , M. Fujii , Y. Ohta , M. Matano , K. Nanki , K. Kawasaki , S. Takahashi , S. Sugimoto , E. Iwasaki , J. Takagi , T. Itoi , M. Kitago , Y. Kitagawa , T. Kanai , T. Sato , Cell Stem Cell 2018, 22, 454. 29337182 10.1016/j.stem.2017.12.009

[16] Y. Miao , A. Ha , W. de Lau , K. Yuki , A. J. M. Santos , C. You , M. H. Geurts , J. Puschhof , C. Pleguezuelos‐Manzano , W. C. Peng , R. Senlice , C. Piani , J. W. Buikema , O. M. Gbenedio , M. Vallon , J. Yuan , S. de Haan , W. Hemrika , K. Rösch , L. T. Dang , D. Baker , M. Ott , P. Depeille , S. M. Wu , J. Drost , R. Nusse , J. P. Roose , J. Piehler , S. F. Boj , C. Y. Janda , et al., Cell Stem Cell 2020, 27, 840. 32818433 10.1016/j.stem.2020.07.020 PMC7655723

[17] H. Chen , C. Lu , B. Ouyang , H. Zhang , Z. Huang , D. Bhatia , S. J. Lee , D. Shah , A. Sura , W. C. Yeh , Y. Li , Cell Chem. Biol. 2020, 27, 598. 32220333 10.1016/j.chembiol.2020.02.009

[18] D. Gumber , M. Do , N. Suresh Kumar , P. R. Sonavane , C. C. N. Wu , L. S. Cruz , S. Grainger , D. Carson , T. Gaasterland , K. Willert , Elife 2020, 9, 63060. 10.7554/eLife.63060 PMC7759383 33331818

[19] Y. Tao , M. Mis , L. Blazer , M. J. Ustav , Z. Steinhart , R. Chidiac , E. Kubarakos , S. O'Brien , X. Wang , N. Jarvik , N. Patel , J. Adams , J. Moffat , S. Angers , S. S. Sidhu , Elife 2019, 8, 46134. 10.7554/eLife.46134 PMC6711705 31452509

[20] S. Seshagiri , E. W. Stawiski , S. Durinck , Z. Modrusan , E. E. Storm , C. B. Conboy , S. Chaudhuri , Y. Guan , V. Janakiraman , B. S. Jaiswal , J. Guillory , C. Ha , G. J. Dijkgraaf , J. Stinson , F. Gnad , M. A. Huntley , J. D. Degenhardt , P. M. Haverty , R. Bourgon , W. Wang , H. Koeppen , R. Gentleman , T. K. Starr , Z. Zhang , D. A. Largaespada , T. D. Wu , F. J. de Sauvage , Nature 2012, 488, 660. 22895193 10.1038/nature11282 PMC3690621

[21] H. X. Hao , Y. Xie , Y. Zhang , O. Charlat , E. Oster , M. Avello , H. Lei , C. Mickanin , D. Liu , H. Ruffner , X. Mao , Q. Ma , R. Zamponi , T. Bouwmeester , P. M. Finan , M. W. Kirschner , J. A. Porter , F. C. Serluca , F. Cong , Nature 2012, 485, 195. 22575959 10.1038/nature11019

[22] B. K. Koo , M. Spit , I. Jordens , T. Y. Low , D. E. Stange , M. van de Wetering , J. H. van Es , S. Mohammed , A. J. Heck , M. M. Maurice , H. Clevers , Nature 2012, 488, 665. 22895187 10.1038/nature11308

[23] N. Fenderico , R. C. van Scherpenzeel , M. Goldflam , D. Proverbio , I. Jordens , T. Kralj , S. Stryeck , T. Z. Bass , G. Hermans , C. Ullman , T. Aastrup , P. Gros , M. M. Maurice , Nat. Commun. 2019, 10, 365. 30664649 10.1038/s41467-018-08172-z PMC6341108

[24] S. A. Ettenberg , O. Charlat , M. P. Daley , S. Liu , K. J. Vincent , D. D. Stuart , A. G. Schuller , J. Yuan , B. Ospina , J. Green , Q. Yu , R. Walsh , S. Li , R. Schmitz , H. Heine , S. Bilic , L. Ostrom , R. Mosher , K. F. Hartlepp , Z. Zhu , S. Fawell , Y. M. Yao , D. Stover , P. M. Finan , J. A. Porter , W. R. Sellers , I. M. Klagge , F. Cong , Proc. Natl. Acad. Sci. 2010, 107, 15473. 20713706 10.1073/pnas.1007428107 PMC2932603

[25] H. Jackson , D. Granger , G. Jones , L. Anderson , S. Friel , D. Rycroft , W. Fieles , J. Tunstead , M. Steward , T. Wattam , A. Walker , J. Griggs , M. Al‐Hajj , C. Shelton , Mol. Cancer Res. 2016, 14, 859. 27401612 10.1158/1541-7786.MCR-16-0088

[26] F. Kawano , H. Suzuki , A. Furuya , M. Sato , Nat. Commun. 2015, 6, 6256. 25708714 10.1038/ncomms7256

[27] P. Tan , L. He , Y. Huang , Y. Zhou , Physiol. Rev. 2022, 102, 1263. 35072525 10.1152/physrev.00021.2021 PMC8993538

[28] L. T. Dang , Y. Miao , A. Ha , K. Yuki , K. Park , C. Y. Janda , K. M. Jude , K. Mohan , N. Ha , M. Vallon , J. Yuan , J. G. Vilches‐Moure , C. J. Kuo , K. C. Garcia , D. Baker , Nat. Struct. Mol. Biol. 2019, 26, 407. 31086346 10.1038/s41594-019-0224-z PMC6582999

[29] A. Levskaya , O. D. Weiner , W. A. Lim , C. A. Voigt , Nature 2009, 461, 997. 19749742 10.1038/nature08446 PMC2989900

[30] L. R. Polstein , M. Juhas , G. Hanna , N. Bursac , C. A. Gersbach , ACS Synth. Biol. 2017, 6, 2003. 28793186 10.1021/acssynbio.7b00147 PMC5767923

[31] W. de Lau , N. Barker , T. Y. Low , B. K. Koo , V. S. Li , H. Teunissen , P. Kujala , A. Haegebarth , P. J. Peters , M. van de Wetering , D. E. Stange , J. E. van Es , D. Guardavaccaro , R. B. Schasfoort , Y. Mohri , K. Nishimori , S. Mohammed , A. J. Heck , H. Clevers , Nature 2011, 476, 293. 21727895 10.1038/nature10337

[32] M. Huch , C. Dorrell , S. F. Boj , J. H. van Es , V. S. Li , M. van de Wetering , T. Sato , K. Hamer , N. Sasaki , M. J. Finegold , A. Haft , R. G. Vries , M. Grompe , H. Clevers , Nature 2013, 494, 247. 23354049 10.1038/nature11826 PMC3634804

[33] T. Sato , R. G. Vries , H. J. Snippert , M. van de Wetering , N. Barker , D. E. Stange , J. H. van Es , A. Abo , P. Kujala , P. J. Peters , H. Clevers , Nature 2009, 459, 262. 19329995 10.1038/nature07935

[34] C. Y. Janda , L. T. Dang , C. You , J. Chang , W. de Lau , Z. A. Zhong , K. S. Yan , O. Marecic , D. Siepe , X. Li , J. D. Moody , B. O. Williams , H. Clevers , J. Piehler , D. Baker , C. J. Kuo , K. C. Garcia , Nature 2017, 545, 234. 28467818 10.1038/nature22306 PMC5815871

[35] G. P. Gao , M. R. Alvira , L. Wang , R. Calcedo , J. Johnston , J. M. Wilson , Proc. Natl. Acad. Sci. 2002, 99, 11854. 12192090 10.1073/pnas.182412299 PMC129358

[36] B. Wang , L. Zhao , M. Fish , C. Y. Logan , R. Nusse , Nature 2015, 524, 180. 26245375 10.1038/nature14863 PMC4589224

[37] Z. Cheng , T. Biechele , Z. Wei , S. Morrone , R. T. Moon , L. Wang , W. Xu , Nat. Struct. Mol. Biol. 2011, 18, 1204. 21984209 10.1038/nsmb.2139 PMC3249237

[38] J. C. Castle , M. Loewer , S. Boegel , J. de Graaf , C. Bender , A. D. Tadmor , V. Boisguerin , T. Bukur , P. Sorn , C. Paret , M. Diken , S. Kreiter , Ö. Türeci , U. Sahin , BMC Genomics 2014, 15, 190. 24621249 10.1186/1471-2164-15-190 PMC4007559

[39] G. Chai , E. Szenker‐Ravi , C. Chung , Z. Li , L. Wang , M. Khatoo , T. Marshall , N. Jiang , X. Yang , J. McEvoy‐Venneri , V. Stanley , P. Anzenberg , N. Lang , V. Wazny , J. Yu , D. M. Virshup , R. Nygaard , F. Mancia , R. Merdzanic , M. B. P. Toralles , P. M. L. Pitanga , R. D. Puri , R. Hernan , W. K. Chung , A. M. Bertoli‐Avella , N. Al‐Sannaa , M. S. Zaki , K. Willert , B. Reversade , J. G. Gleeson , N. Engl. J. Med. 2021, 385, 1292. 34587386 10.1056/NEJMoa2033911 PMC9017221

[40] J. Liu , Q. Xiao , J. Xiao , C. Niu , Y. Li , X. Zhang , Z. Zhou , G. Shu , G. Yin , Sig. Transduct. Target Ther. 2022, 7, 3. 10.1038/s41392-021-00762-6 PMC8724284 34980884

[41] H. Hirai , K. Matoba , E. Mihara , T. Arimori , J. Takagi , Nat. Struct. Mol. Biol. 2019, 26, 372. 31036956 10.1038/s41594-019-0216-z

[42] A. M. Vargason , A. C. Anselmo , S. Mitragotri , Nat. Biomed. Eng. 2021, 5, 951. 33795852 10.1038/s41551-021-00698-w

[43] N. Barker , M. Huch , P. Kujala , M. van de Wetering , H. J. Snippert , J. H. van Es , T. Sato , D. E. Stange , H. Begthel , M. van den Born , E. Danenberg , S. van den Brink , J. Korving , A. Abo , P. J. Peters , N. Wright , R. Poulsom , H. Clevers , Cell Stem Cell 2010, 6, 25. 20085740 10.1016/j.stem.2009.11.013

[44] H. Hu , H. Gehart , B. Artegiani , C. LÖpez‐Iglesias , F. Dekkers , O. Basak , J. van Es , S. M. Chuva de Sousa Lopes , H. Begthel , J. Korving , M. van den Born , C. Zou , C. Quirk , L. Chiriboga , C. M. Rice , S. Ma , A. Rios , P. J. Peters , Y. P. de Jong , H. Clevers , Cell 2018, 175, 1591. 30500538 10.1016/j.cell.2018.11.013

[45] F. J. M. Roos , G. S. van Tienderen , H. Wu , I. Bordeu , D. Vinke , L. M. Albarinos , K. Monfils , S. Niesten , R. Smits , J. Willemse , O. Rosmark , G. Westergren‐Thorsson , D. J. Kunz , M. de Wit , P. J. French , L. Vallier , I. J. JNM , R. Bartfai , H. Marks , B. D. Simons , M. E. van Royen , M. M. A. Verstegen , L. J. W. van der Laan , Cell Stem Cell 2022, 29, 776. 35523140 10.1016/j.stem.2022.04.011

[46] A. Waterhouse , M. Bertoni , S. Bienert , G. Studer , G. Tauriello , R. Gumienny , F. T. Heer , T. A. P. de Beer , C. Rempfer , L. Bordoli , R. Lepore , T. Schwede , Nucleic Acids Res. 2018, 46, W296. 29788355 10.1093/nar/gky427 PMC6030848

