# Supplementary material for: Manipulation of Wnt/β‐Catenin Signaling by Synthetic Frizzled Agonist and LRP Antagonist in Organoid Cultures and In Vivo
Source: Small Methods. 2025 Mar 28;10(2):2500425. doi: 10.1002/smtd.202500425 (PMC12825328; doi:10.1002/smtd.202500425)
Supplement: Supplementary file 1 — Supporting Information [file SMTD-10-2500425-s001.docx]

Supporting Information

**Manipulation of Wnt/β-catenin Signaling by Synthetic Frizzled Agonist and LRP Antagonist in Organoid Cultures and *In Vivo***

*Quanhui Dai, Jiawen Wang, Zihuan Lin, Danni Yu, Hui Yang, Jinsong Wei, Xiaoyu Li, Hao Hu, Chao Ni, and Bing Zhao^*^*

Figure S1-S10

Table S1-S2

**
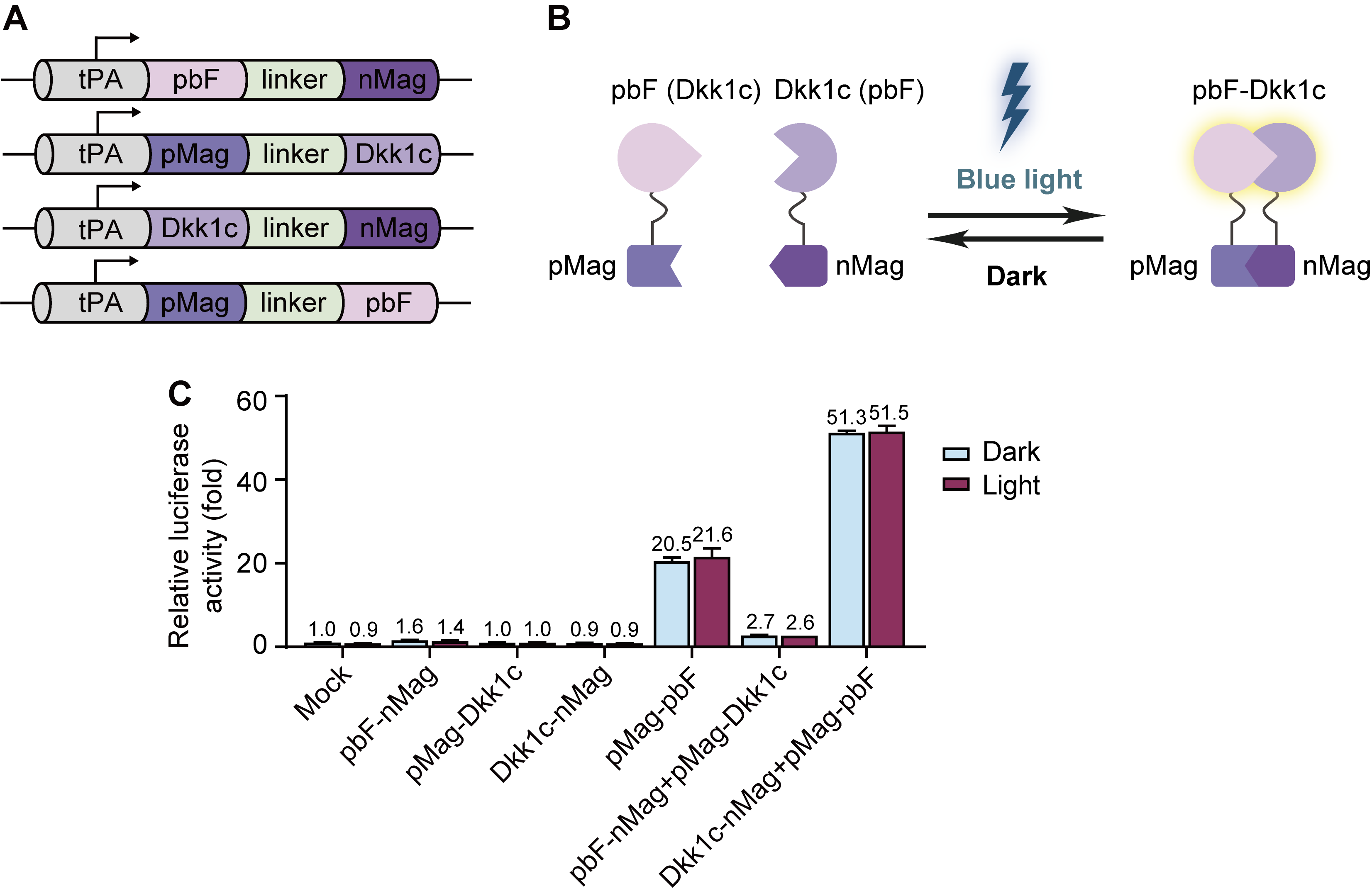
**

**Figure S1. pMag-pbF activates Wnt/β-catenin signaling without blue light illumination.** A) Scheme depicting combinations of variant sWnt fragments (pbF/Dkk1c) with the pMag/nMag. B) Schematic diagram of light-induced sWnt for controlled Wnt activation. pMag is tagged with a sWnt fragment, and nMag is tagged with the other sWnt fragment. These fusions are monomers in the dark state. Upon blue light irradiation, dimerization of Magnets leads to the complementation of pbF and Dkk1c. C) Luciferase reporter assay for evaluating TOP-Flash signal in HEK 293T cells overexpressing pbF-nMag, pMag-Dkk1c, Dkk1c-nMag, pMag-pbF, and their combinations, with or without illumination. Data represent mean ± SD; *n* = 3 independent experiments.


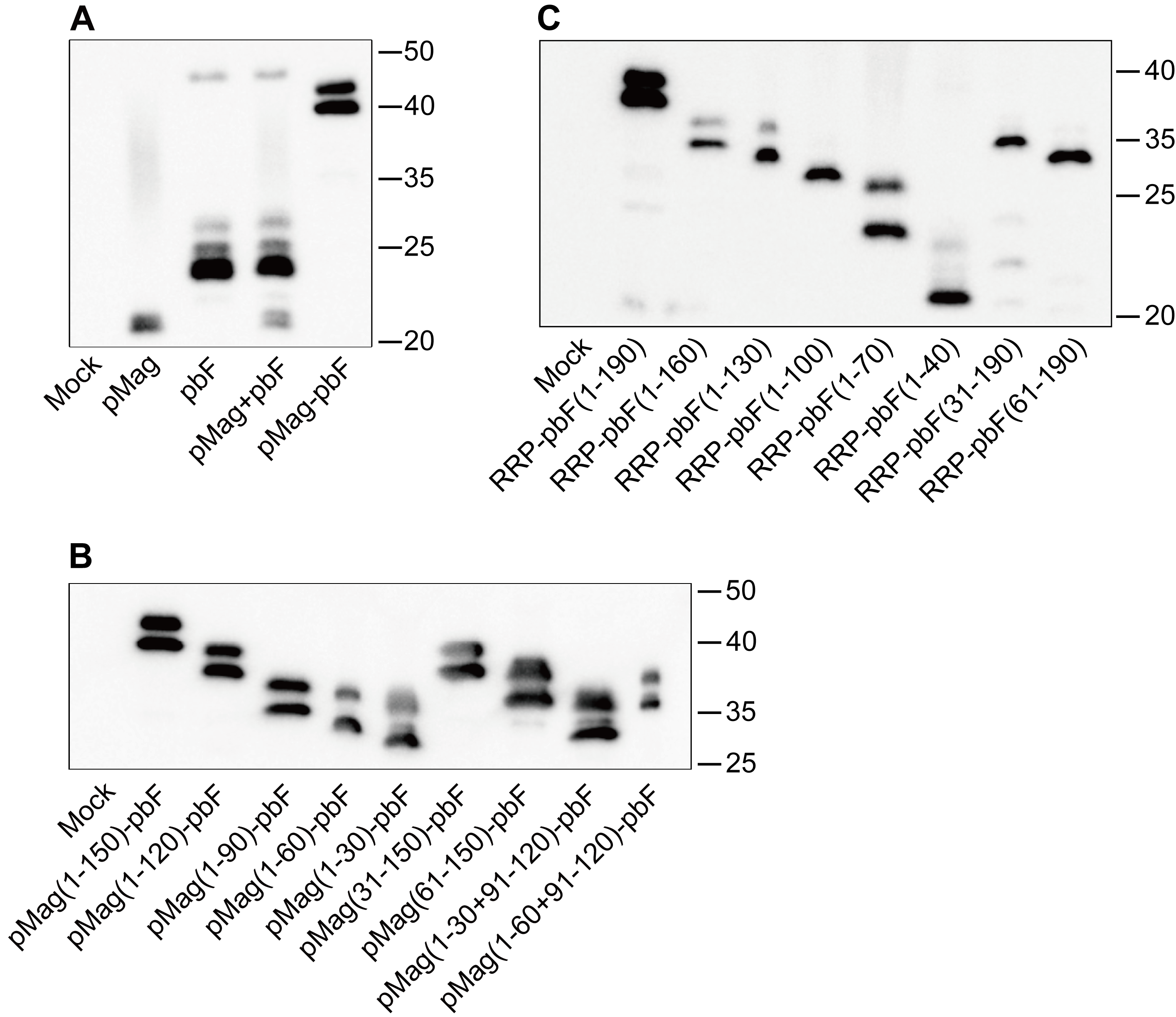


**Figure S2. Western blot analysis of the expression of proteins.** A) Detection of the expression in supernatant after overexpressing indicated proteins by Western blot for anti-Myc. B) Detection of the expression of proteins in supernatant after overexpressing truncated variants of pMag fusion with pbF for anti-Myc. C) Detection of expression of proteins from truncated variants of pbF fusion with RRP in supernatant after overexpression by Western blot for anti-Myc.

**
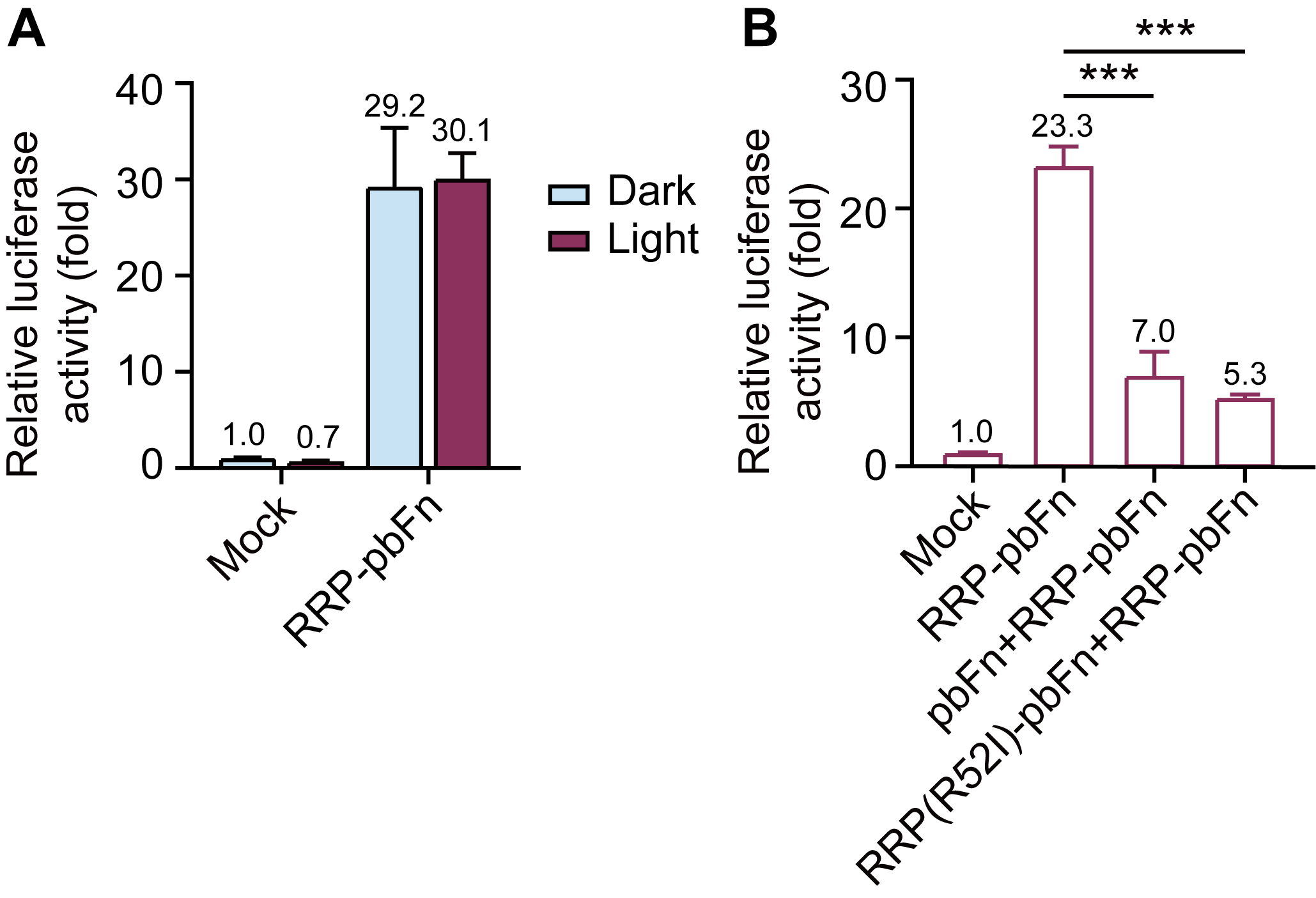
**

**Figure S3. RRP(R52I)-pbFn blocks the Wnt signaling activation induced by RRP-pbFn.** A) Luciferase reporter assay for evaluating TOP-Flash signal in HEK 293T cells overexpressing RRP-pbFn with or without illumination. B) Luciferase reporter assay for evaluating TOP-Flash signal in HEK 293T cells overexpressing RRP-pbFn, and combinations of RRP-pbFn with pbFn or RRP(R52I)-pbFn, respectively. TOP-Flash data represent mean ± SD; *n* = 3 independent experiments. **p* < 0.05; ***p* < 0.01; ****p* < 0.001.


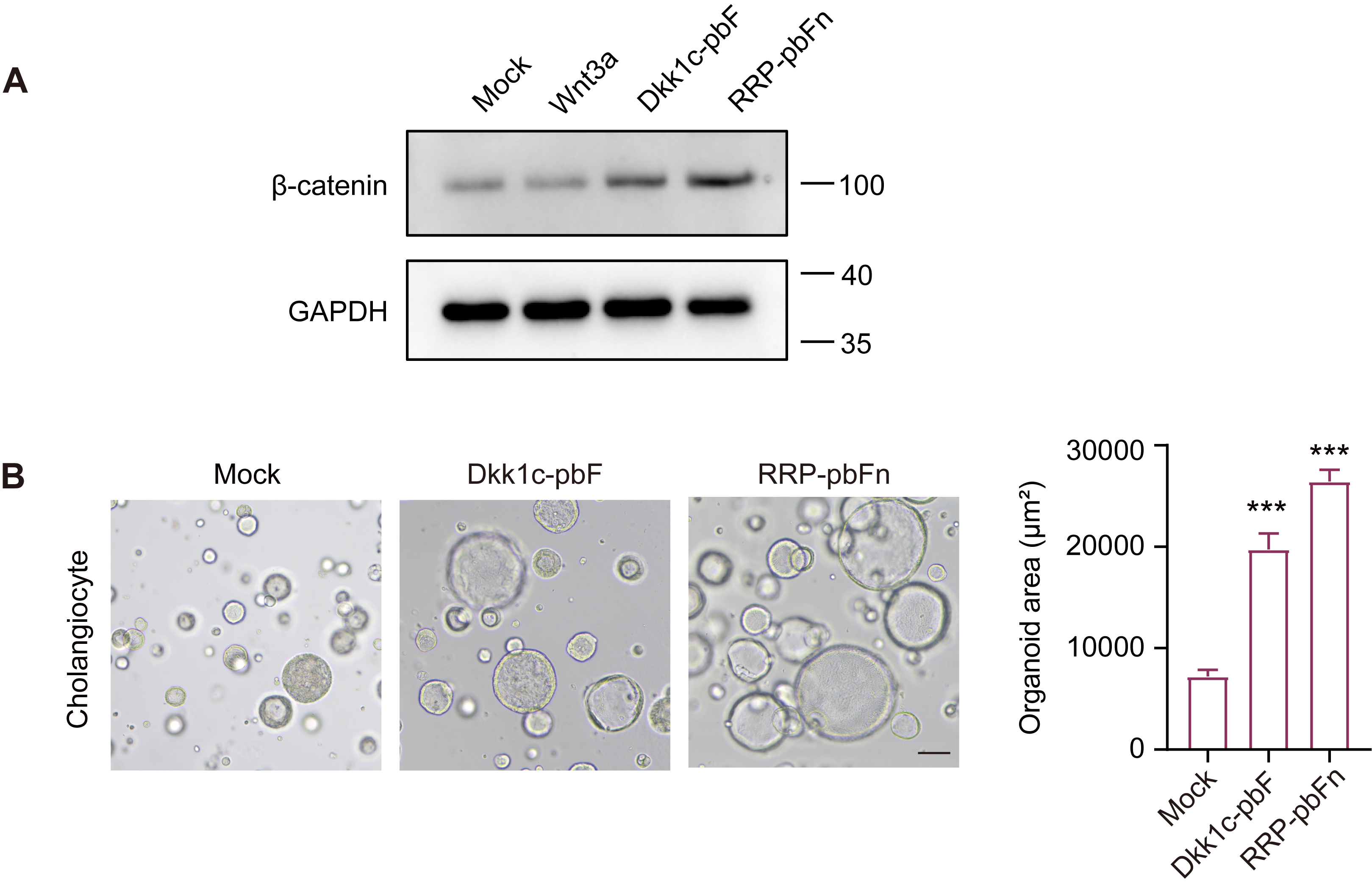


**Figure S4. The comparison between Dkk1c-pbF and RRP-pbFn.** A) Western blot analysis of β-catenin induced by recombinant Wnt3a, Dkk1c-pbF and RRP-pbFn. B) Left: representative bright-field images of cholangiocyte organoids expanded in culture media supplemented with 200 ng/mL Dkk1c-pbF or RRP-pbFn. The scale bar represents 100 μm. Right: quantification of the organoid area. Data represent mean ± SD, and *n* = 3. ****p* < 0.001.

**
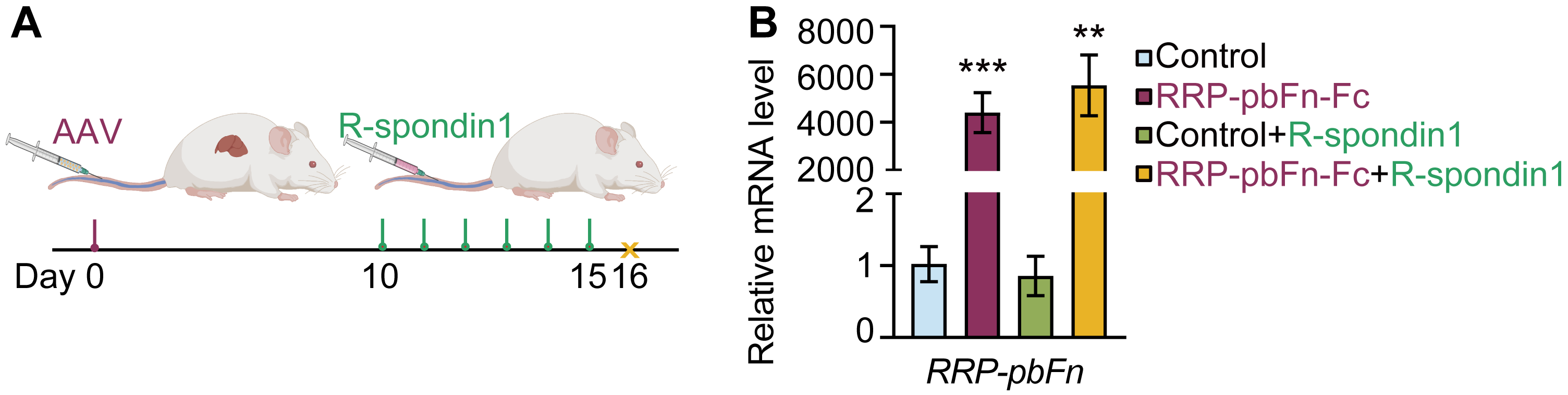
**

**Figure S5. AAV-directed expression of RRP-pbFn-Fc in mice.** A) Scheme depicting intravenous injection of AAV and R-spondin1. B) qRT-PCR validation of AAV-mediated transgene RRP-pbFn-Fc expression in mouse liver. Data represent mean ± SD. *n* = 3 mice per group. **p* < 0.05; ***p* < 0.01; ****p* < 0.001.

**
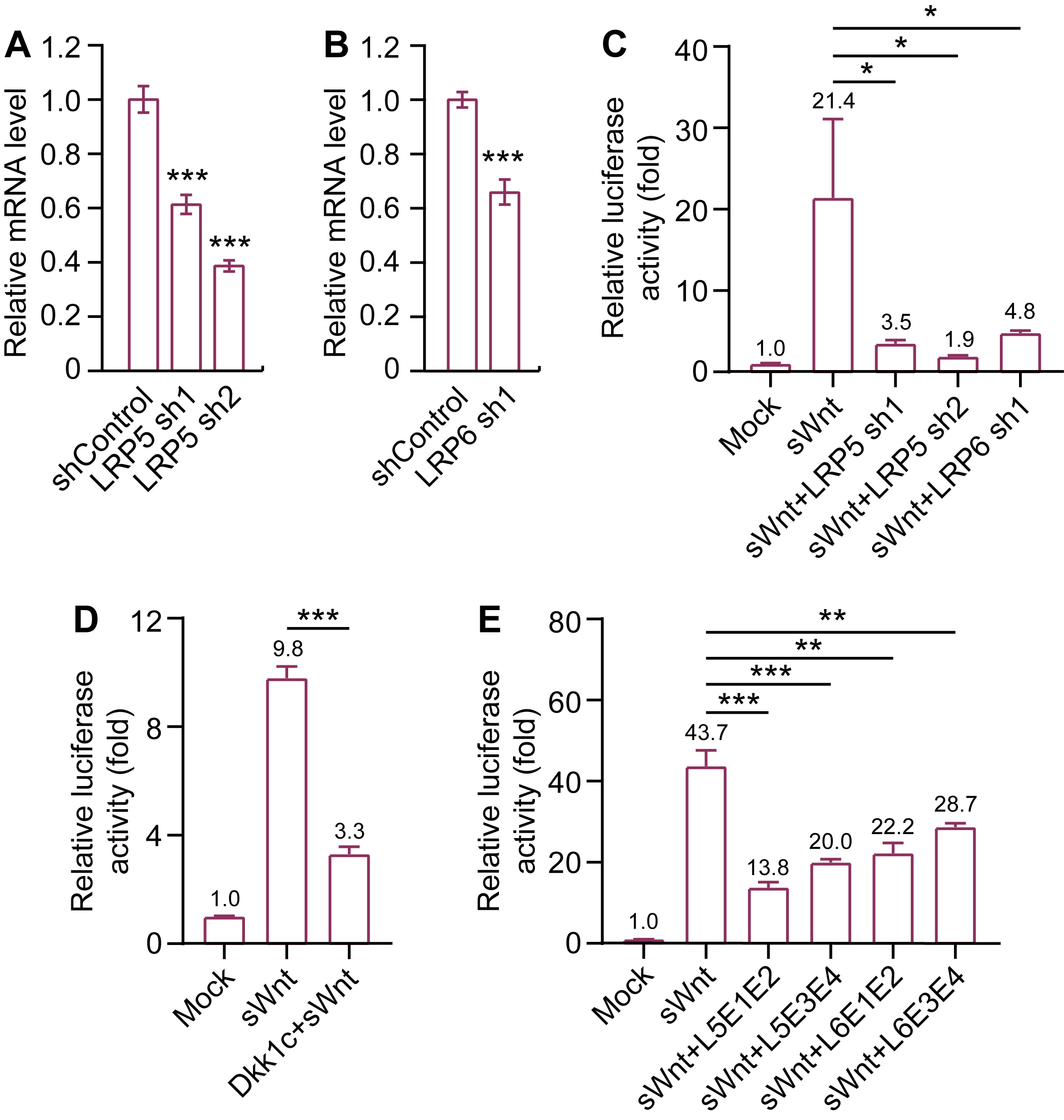
**

**Figure S6. Blocking the function of LRP5/6 prevents sWnt-induced Wnt signaling.** A, B) qRT-PCR analysis of the expression of *LRP5* (A) or *LRP6* (B) in HEK 293T cells after interference with LRP5 shRNAs, LRP6 shRNA, or scrambled shRNA (shControl). C) TOP-Flash luciferase reporter assay showing the Wnt activation induced by 50 ng/mL sWnt alone and in combination with overexpressing the indicated shRNAs. D) TOP-Flash luciferase reporter assay showing the Wnt activation induced by 50 ng/mL sWnt alone and in combination with overexpression of Dkk1c. E) TOP-Flash luciferase reporter assay showing the impact of L5E1E2, L5E3E4, L6E1E2, or L6E3E4 on 50 ng/mL sWnt mediated activation of Wnt signaling. Data represent mean ± SD; *n* = 3 independent experiments. **p* < 0.05; ***p* < 0.01; ****p* < 0.001.

**
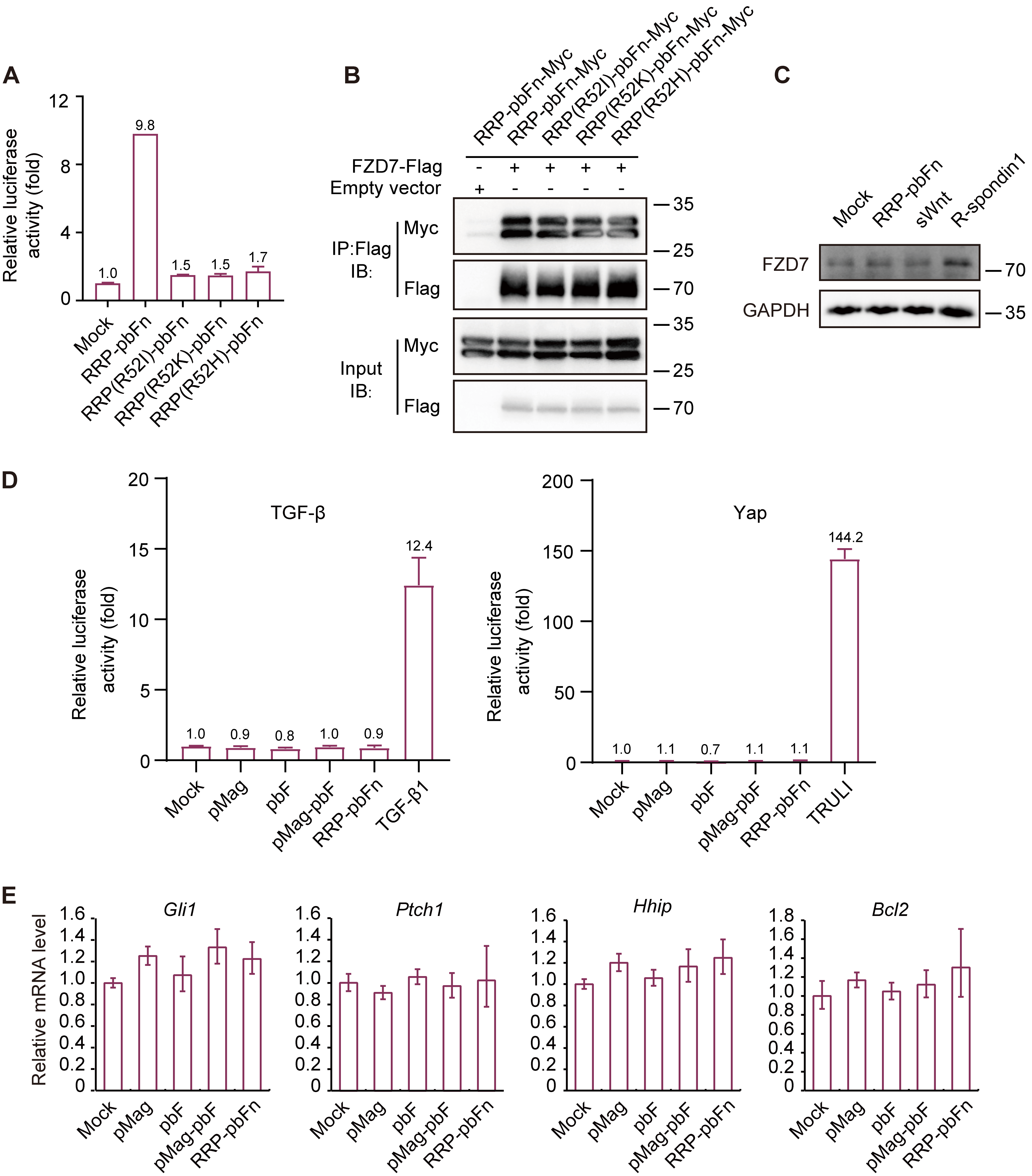
**

**Figure S7. Analyses of the interaction and function of variants of RRP-pbFn.** A) TOP-Flash luciferase reporter assay showing the Wnt activation induced by RRP-pbFn, RRP(R52I)-pbFn, RRP(R52K)-pbFn, RRP(R52H)-pbFn. TOP-Flash data represent mean ± SD; *n* = 3 independent experiments. **p* < 0.05; ***p* < 0.01; ****p* < 0.001. B) Co-IP analysis of the interaction between variants of RRP-pbFn and FZD7. Assays were performed in HEK 293T cells transfected with variants of RRP-pbFn-Myc and FZD7-Flag followed by immunoprecipitation with Flag-beads. C) Western blot analysis of FZD7 from HEK 293T cell membrane proteins induced by RRP-pbFn, sWnt and R-spondin1. D) Luciferase reporter assay showing signaling activity induced by overexpression of pMag, pMag-pbFn, RRP-pbFn, and positive control (0.1 nM TGF-β1 or 10 μM TRULI). Data represent mean ± SD; *n* = 3 independent experiments. E) qRT-PCR analysis of Smo pathways target genes induced by overexpression of pMag, pbF, pMag-pbFn and RRP-pbFn. Data represent mean ± SD; *n* = 3 independent experiments.

**
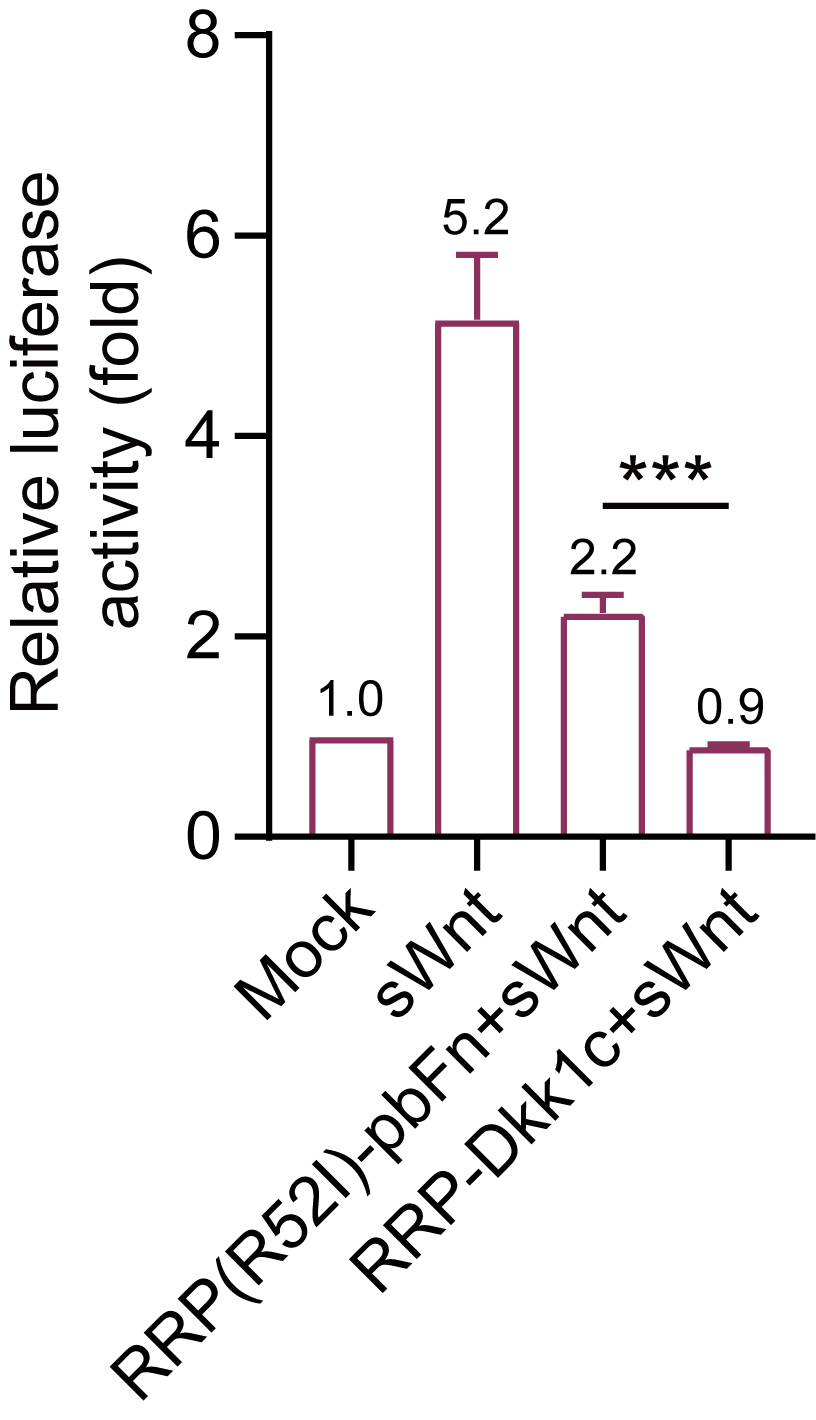
**

**Figure S8. RRP-Dkk1c blocks the Wnt signaling activation induced by sWnt.** TOP-Flash luciferase reporter assay for evaluating Wnt activation in HEK 293T cells induced by 50 ng/mL sWnt alone and in combination with overexpressing RRP(R52I)-pbFn or RRP-Dkk1c. Data represent mean ± SD; *n* = 3 independent experiments. **p* < 0.05; ***p* < 0.01; ****p* < 0.001.

**
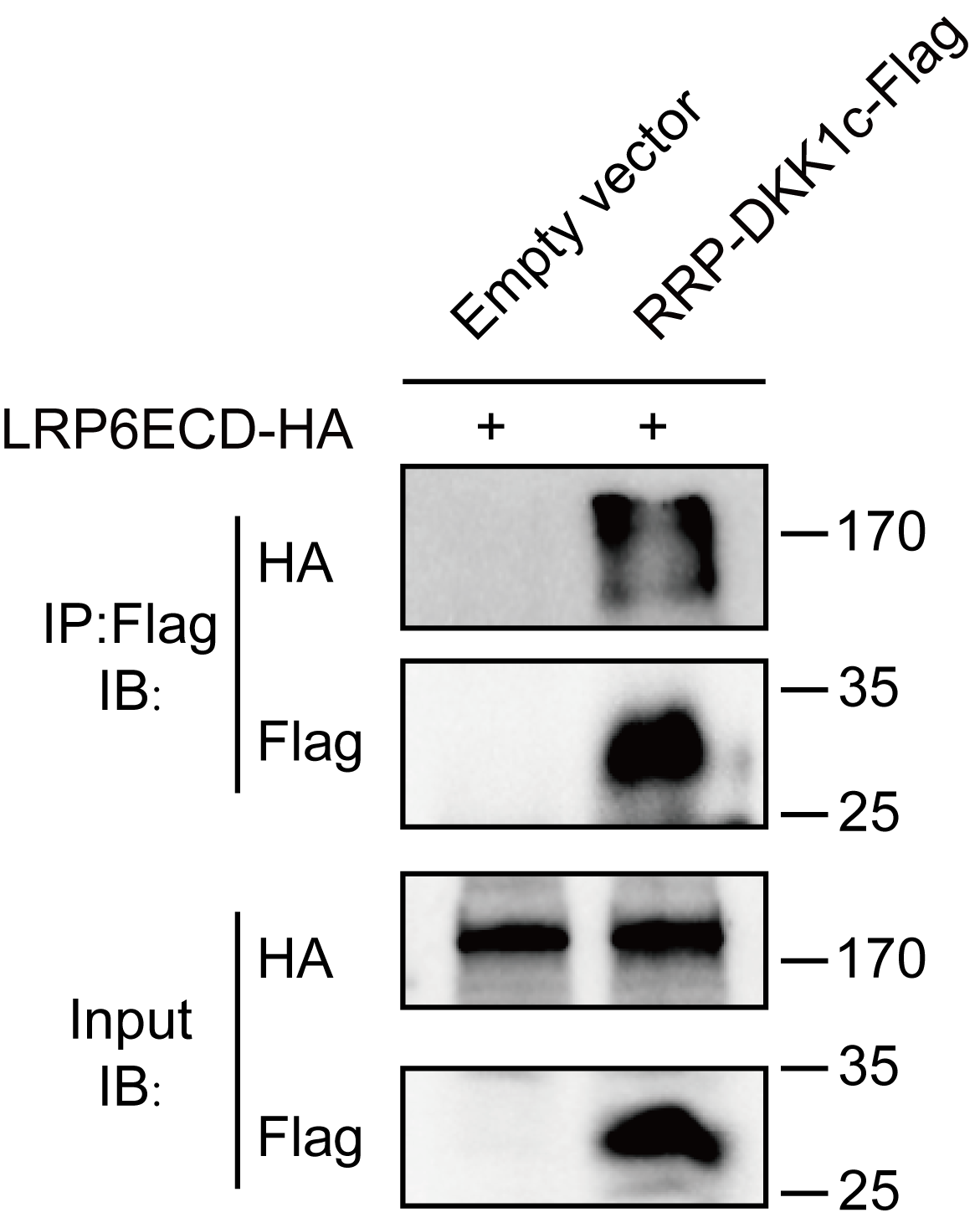
**

**Figure S9. Co-IP analysis of the interaction between RRP-Dkk1c and LRP6 ectodomain.** Assays were performed in HEK 293T cells transfected with RRP-Dkk1c-Flag and LRP6ECD-HA, and Flag-tagged proteins were immunoprecipitation with Flag-beads.

**
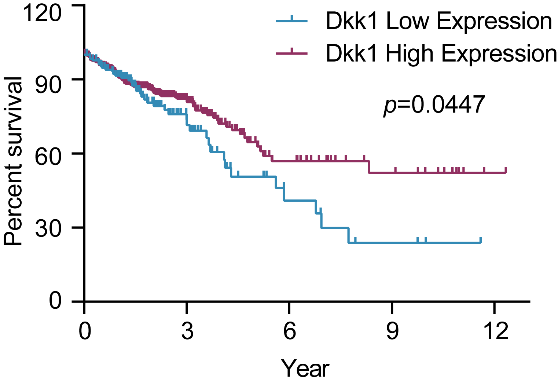
**

**Figure S10. Kaplan-Meier estimates survival probability by Dkk1 expression level in colorectal cancer.** Overall survival rate of colorectal cancer patients according to different expression level of Dkk1. The endpoint was 13 years. Survival curves were compared via Log-rank test and visualized by Kaplan-Meier method.

**Table S1. Primers for shRNA**

| Name | Sequence (5’-3’) |
| --- | --- |
| *Lrp5* sh1 | 5’-CATGATCGAGTCGTCCAAC-3’ |
| *Lrp5* sh2 | 5’-TCATTGATCTCAGTGTTCACA-3’ |
| *Lrp6* sh1 | 5’-CCGCATGGTGATTGATGA-3’ |
| scrambled shRNA | 5’-CAACAAGATGAAGAGCACCAA-3’ |

**Table S2. Primers for RT-qPCR.**

| Quantitative RT-PCR primers | Forward (5'-3') | Reverse (5'-3') |
| --- | --- | --- |
| h-*LRP5* | ACTCGCTGTGAGGAGGACAAT | GGCAGGCGCATGTGTAGAA |
| h-*LRP6* | TTTATGCAAACAGACGGGACTT | GCCTCCAACTACAATCGTAGC |
| h-*Lgr5* | GTGGACTGCTCCGACCTG | GCTGACTGATGTTGTTCATACTGAG |
| h-*Axin2* | CAACACCAGGCGGAACGAA | GCCCAATAAGGAGTGTAAGGACT |
| h-*Gapdh* | GAAGGTGAAGGTCGGAGTC | GAAGATGGTGATGGGATTTC |
| m-*Lgr5* | CGGGACCTTGAAGATTTCCT | GATTCGGATCAGCCAGCTAC |
| m-*Axin2* | TGACTCTCCTTCCAGATCCCA | TGCCCACACTAGGCTGACA |
| m-*Cyp2f2* | CCGGAACTTTGGAGGCATGAA | GGTCATCAGCAGGGTATCCAT |
| m-*GS* | TGAACAAAGGCATCAAGCAAATG | CAGTCCAGGGTACGGGTCTT |
| m-*Gapdh* | AGGTCGGTGTGAACGGATTTG | TGTAGACCATGTAGTTGAGGTCA |
| *RRP-pbF* | TGATTAGCAAAGGAGCCGATG | TTGACCACTTCCTTATGACCG |
